# G1/S restriction point coordinates phasic gene expression and cell differentiation

**DOI:** 10.1038/s41467-022-31101-0

**Published:** 2022-06-27

**Authors:** Brian DeVeale, Leqian Liu, Ryan Boileau, Jennifer Swindlehurst-Chan, Bryan Marsh, Jacob W. Freimer, Adam Abate, Robert Blelloch

**Affiliations:** 1 The Eli and Edythe Broad Center of Regeneration Medicine and Stem Cell Research, Center for Reproductive Sciences, University of California San Francisco, San Francisco, CA USA; 2 Department of Urology, University of California San Francisco, San Francisco, CA USA; 3 Department of Bioengineering and Therapeutic Sciences, University of California San Francisco, San Francisco, CA USA; 4 California Institute for Quantitative Biosciences, University of California San Francisco, San Francisco, CA USA; 5 Chan Zuckerberg Biohub, San Francisco, CA USA

**Keywords:** Checkpoints, Gastrulation, Embryonic stem cells

## Abstract

Pluripotent embryonic stem cells have a unique cell cycle structure with a suppressed G1/S restriction point and little differential expression across the cell cycle phases. Here, we evaluate the link between G1/S restriction point activation, phasic gene expression, and cellular differentiation. Expression analysis reveals a gain in phasic gene expression across lineages between embryonic days E7.5 and E9.5. Genetic manipulation of the G1/S restriction point regulators miR-302 and P27 respectively accelerates or delays the onset of phasic gene expression in mouse embryos. Loss of miR-302-mediated p21 or p27 suppression expedites embryonic stem cell differentiation, while a constitutive Cyclin E mutant blocks it. Together, these findings uncover a causal relationship between emergence of the G1/S restriction point with a gain in phasic gene expression and cellular differentiation.

## Abstract (web summary)

Somatic cells display phasic gene expression with compartmentalized gene expression during the cell cycle, while pluripotent cells lack phasic expression. Here, the authors describe a pathway linking microRNA regulation of the G1/S restriction point with phasic gene expression and cell differentiation during mammalian development.

## Introduction

The cell cycle differs greatly between early embryonic cells and the proliferating somatic cells of later development. In pluripotent mouse embryonic stem cells (ESCs) derived from the peri-implantation epiblast, the G1 phase is abridged, the G1/S restriction point is suppressed, and the CyclinE/Cylin-Dependent Kinase 2 (CDK2) complex is constitutively active through all cell cycle phases^1–4^. In contrast, in somatic cells, there is an extended G1, a functional G1/S restriction point, and a CyclinE/CDK2 complex that shows differential activity across phases with maximum activity at the G1/S transition^3–5^. Therefore, the cell cycle must be remodeled during development.

Along with the unusual cell cycle structure, pluripotent stem cells show minimal differential expression between cell cycle phases^6,7^. In contrast, somatic cells show phasic expression of up to 20% of all expressed genes^8–13^. Phasic expression compartmentalizes discrete processes in the cell cycle such as DNA synthesis and mitosis^14^, but can also regulate cell fate decisions^15,16^. Therefore, suppression of extensive phasic expression may be a means to inhibit premature differentiation of pluripotent cells.

Here, we study the potential link between cell cycle structure, phasic gene expression, and cell differentiation in the developing mouse embryo from pluripotency to early organogenesis. We reveal an increase in phasic gene expression during embryogenesis associated with decreasing levels of the microRNA, miR-302, and the formation of G1/S restriction point. Loss of miR-302 results in premature gain in phasic expression while loss of its target, P27, results in a delay in phasic expression in vivo, prior to the premature differentiation phenotype of the neuroectoderm seen in miR-302 knockouts. Similarly, in cell culture, loss of miR-302 or of its target sites in the p21 or p27 3′UTR results in premature differentiation, while constitutive Cyclin E1 blocks differentiation. These findings link the gain of the G1/S restriction point with phasic gene expression and cellular differentiation.

## Results

### Gain of phasic gene expression during embryogenesis

To evaluate when cell cycle regulated gene expression increases during normal mouse development, we sought an assay capable of resolving phasic expression in low input samples. We sorted mouse 3T3 somatic cells and ESCs into G1 and G2/M based on DNA content and performed 3’ RNA- sequencing (Supplementary Fig. 1A). Differential expression analysis between G1 and G2/M revealed that most genes, such as the housekeeper *Actb* were not phasically expressed in either cell line (Supplementary Fig. 1B), and canonically phasic genes, such as *Prc1* and *Cdc6* were phasically expressed exclusively in 3T3 cells (Supplementary Fig. 1C). Overall, differential expression analysis between G1 and G2/M showed that, as expected, phasic expression was much more pronounced in 3T3 somatic cells than pluripotent ESCs (Supplementary Fig. 1D–J, Supplementary Data 1). The genes phasically expressed in 3T3 cells and not in ESCs included mitosis-associated proteins such as *Ccnb1, Aurka*, and *Plk1* as well as G1/S regulators such as *Cdc6, E2f1* and *Ccne1*. Overall, the phasically expressed genes were enriched for genes identified as phasic in other contexts and for cell cycle related functions (81 Cyclebase genes among 167 total, 25-fold enrichment, hypergeometric *P* = 1.34e−96, Supplementary Fig. 1K).

**Fig. 1:**
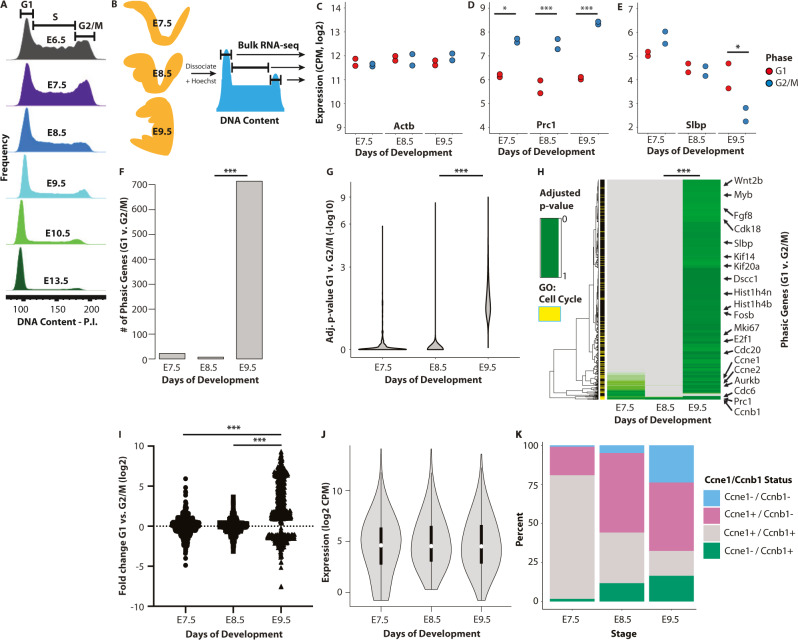
Gain of phasic expression following gastrulation. **A** DNA-content analysis of cells from whole embryos ranging from embryonic day 6.5 (E6.5) to E13.5 (representative litters, *n* = 2 biologically independent samples for each stage, embryos from each litter were pooled). **B** Schematic of the data collection and analysis. E7.5, E8.5, and E9.5 embryos were dissociated to single cells, sorted by cell cycle phase based on DNA-content, and subjected to RNA-sequencing for differential expression analysis between the phases at each timepoint. **C**–**E** The expression of *Actb* (**C**), *Prc1* (adjusted *P* value (adj. *P*), *P = 0.029 [E7.5]; ***P = 0.00017 [E8.5]; ***P = 1.02e−9 [E9.5]) (**D**) and *Slbp* (* adj. *P* = 0.05) (**E**) at each developmental stage. *n* = 2 biologically independent litters for each stage and phase. Litters of 6 and 4 embryos at E7.5, 8 and 6 embryos at E8.5, and 3 and 5 embryos at E9.5 were pooled. **F** The number of genes showing differential expression between G1 and G2/M (adj. *P* < 0.1) increases between E7.5 and E9.5 (***P < 0.001, Chi-square test). **G** The distribution of adjusted *P* values at each developmental stage for union of all genes identified in F (***P < 0.001, Wilcoxon Rank Test, two-sided). **H** Heat map of *P* values for all genes shown in G (***P < 0.001, Wilcoxon Rank Test, two-sided). **I** The distribution of fold-change values for all genes shown in G (***P < 0.001, Tukey’s post hoc, one-way ANOVA). **J** The distribution of log2 counts per million for all genes shown in (**G**) (no significant change, *P* > 0.5, one-way ANOVA, n = 714 genes). White dot, median. Box edges, 25th and 75th quartiles. Whiskers, 1.5× the IQR of the box edge. **K** The relative distribution of Cyclin E1 and Cyclin B1 protein in E7.5, E8.5, and E9.5 embryos. The fraction of cells expressing both Cyclin E1 and Cyclin B1 declines between E7.5 and E9.5 (**P < 0.01, one-way ANOVA; *n* = 521 (E7.5), *n* = 680 (E8.5), *n* = 1516 (E9.5) biologically independent cells). **C**–**H** Differential expression was evaluated with a Wald Chi-squared test and adjusted for multiple tests using the Benjamini-Hochberg (BH) approach, *n* = 2 biologically independent samples for each stage and phase (each dot represents an individual sample in **C**–**E**). The Source Data for (**C**–**E**, **K**) are provided in “Source Data.xlsx”.

Having validated the approach, we next asked if and when phasic expression is gained in vivo. To narrow down the developmental window to evaluate, we hypothesized that phasic expression would likely coincide with the establishment of a more somatic cell-like cell cycle structure. DNA-content flow cytometry of embryos ranging from the pluripotent epiblast stage (embryo day (E) 6.5) to mid-gestation (E13.5) showed a major shift toward a somatic cell-like cycle structure between E7.5 and E9.5 (Fig. 1A, Supplementary Fig. 2A–C). G1 and G2/M cells from E7.5, E8.5, and E9.5 embryos were dissected away from extra-embryonic tissues, sorted and RNA sequenced (Fig. 1B, Supplementary Fig. 2D, E). Differential expression analysis discerned three patterns of phasic expression across the three embryonic stages: non-phasic at all stages as exemplified by *Actb* (Fig. 1C), phasic at all stages as exemplified by *Prc1* (Fig. 1D), and stage specific phasic expression as exemplified by *Slbp* (Fig. 1E). The number of genes that were constitutively phasic was small and included mostly G2/M regulators such as *Prc1, Aurka*, and *Ube2c* or mitosis-associated proteins such as *Cenpf* and *Nusap1*. However, there was a large gain from E8.5 to E9.5 in the number of genes showing significant differential phasic expression (Fig. 1F–I, Supplementary Fig. 2F, G, Supplementary Data 2). The gain occurred among genes spanning a large range of expression values and the average expression values across the cell cycle remained relatively constant even though the differential between phases increased (Fig. 1J, Supplementary Fig. 2H). The genes that became phasically expressed included additional G2/M regulators along with classic G1/S genes such as *E2f1*, *Slbp*, and *Pcna* (Supplementary Data 2)^17^. Overall, the genes that became phasic by E9.5 were enriched for genes identified as phasic in other contexts and for cell cycle related functions (61 Cyclebase genes among 714 total, 5-fold enrichment, hypergeometric *P* = 3.91e−25, Supplementary Fig. 2I–N)^18^.

To determine whether this gain was due to changes in phasic transcription or post-transcriptional mechanisms such as RNA stability relative to cell cycle length, we repeated these experiments on independent E8.5-E9.5 embryos using a total RNA-sequencing approach allowing the capture of introns as well as exons. Introns are rapidly degraded and thus their levels correlate closely with their transcription at the time of RNA capture^19^. Analysis of the resulting intronic and exonic data showed agreement between the two with a highly significant gain in phasic expression and transcription between E8.5 and E9.5 (Supplementary Fig. 2O, Supplementary Data 3). Overall differential exonic and intronic abundance across G1 and G2/M were highly correlated among all phasically expressed genes (Supplementary Fig. 2P, *r* = 0.84). Among genes where intronic data resolved phasic transcription and/or stability as the source of phasic expression, linear models revealed that differential transcription (Δ exon & Δ intron) was responsible for 277 phasically expressed genes, differential stability (Δ exons, not Δ introns) for 9 phasic genes, and a combination of the two was responsible for 11 genes. Therefore, the gain of phasic expression seen from E8.5 to E9.5 was driven primarily by a gain in phasic transcription and not phasic stability.

Next, we asked if this phasic expression was reflected in protein levels. We focused on two classic cell cycle regulatory proteins, Cyclin E1 and Cyclin B1. Cyclin E1 mRNA levels showed a clear gain in phasic expression between E7.5 and E9.5 with an enrichment in G1, while phasic expression of Cyclin B1 mRNA became increasingly enriched in G2M (Supplementary Fig. 3A, B). To evaluate how this change was reflected in protein levels, we performed co-staining for the two proteins in E7.5, E8.5, and E9.5 embryos. At E7.5, 79.1% of cells were double positive (Fig. 1K, Supplementary Fig. 3C). Over developmental time the co-expression of these two proteins resolved such that only 15.9% were double positive at E9.5 (Fig. 1K, Supplementary Fig. 3D, E). Together, these data show a striking pattern of gained phasic expression from E7.5 to E9.5 in mouse embryos.

### miR-302/P27 regulates timing of phasic transcription

The redistribution of S-phase cells to G1 between E7.5 and E9.5 embryos is consistent with G1/S restriction point establishment between these stages. Therefore, we considered the possibility that the G1/S restriction point may regulate phasic expression. MicroRNAs of the miR-302 family suppress the G1/S restriction point^20,21^ and are expressed in the pluripotent epiblast at E5.5 before decreasing with varied timing across all germ layers, including ectoderm, by E9.5^22,23^. Small RNA sequencing showed that miR-302 family miRNAs are the most abundant family at E7.5 (18.8%), before declining to 1.0% at E9.5 (Supplementary Fig. 4A–C). This decline in miR-302 abundance coincided with the developmental window in which phasic expression was gained.

**Fig. 2:**
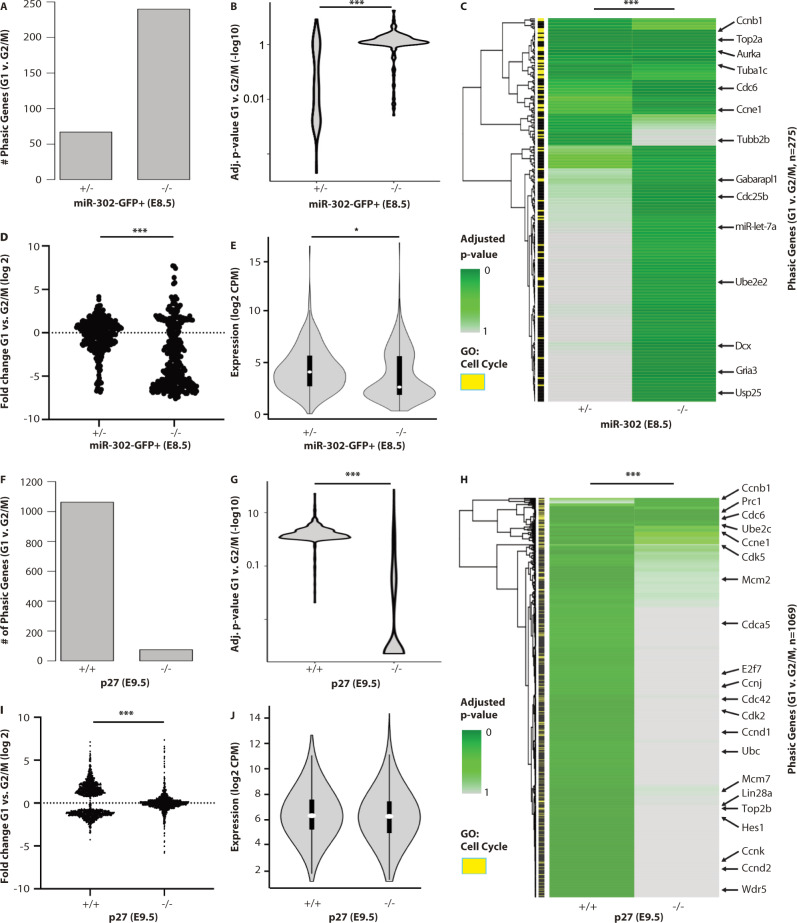
*miR-302* suppresses while p27 promotes the onset of phasic expression. **A** The number of genes showing differential expression between G1 and G2/M (BH adj. *P* < 0.1) in *mir-302*-GFP+ sorted populations from control and *mir-302*−/− E8.5 embryos (*n* = 3 biologically independent embryos). **B** The distribution of adjusted *P* values of the union of all genes identified in (**F**) (Wilcoxon Rank Test, two-sided, ***P < 0.001). **C** A heat map of *P* values for all genes shown in (**B**) (Wilcoxon Rank Test, two-sided, ***P < 0.001). **D** The distribution of fold-change values for all genes shown in (**C**) (***P < 0.001, two-tailed *t* test). **E** The distribution of log2 counts per million for all gene shown in (**G**) (*P* < 0.05, two-tailed *t* test, *n* = 284 genes). White dot, median. Box edges, 25th and 75th quartiles. Whiskers, 1.5× the IQR of the box edge. **F**–**H** Same as (**A**–**E**), except comparing *p27* control to knockouts (*n* = 3 biologically independent embryos). **I** ***P < 0.001, two-tailed *t* test (**J**) no significant change, *P* > 0.5, two-tailed *t* test, *n* = 1069 genes. Differential expression was evaluated with a Wald Chi-squared test (**A**–**C**, **F**–**H**).

To ask if *mir-302* regulated the onset of phasic expression, we compared differential gene expression between G1 and G2/M in *mir-302*−/− and control (*mir-302*+/−) embryos. We made the comparison at E8.5, an intermediate stage in the onset of periodic transcription. We sorted cells with active *mir-302* loci using a green fluorescent protein (GFP) reporter that had been knocked into the mutated allele (Supplementary Fig. 4D)^22^. As expected, *mir-302* target genes were upregulated in knockouts (Supplementary Fig. 4E). Differential expression between G1 and G2/M showed a large gain in phasic expression in the sorted *mir-302* knockout versus control E8.5 cells (Fig. 2A–D, Supplementary Fig. 4F, Supplementary Data 4). The gain in phasic expression was not secondary to an increase in overall expression of the phasic genes (Fig. 2E), but did coincide with a slight increase in G1 cells (Supplementary Fig. 4G). These data show *mir-302* regulates phasic expression in vivo.

To explore whether *mir-302* and phasic expression are linked by the G1/S restriction point, we asked if *mir-302* was impacting classic G1/S restriction point regulators during the E7.5 to E9.5 transition. The precociously expressed phasic genes in *mir-302* knockouts were depleted for *mir-302* targets, consistent with an indirect mechanism (Supplementary Data 4, *P* = 0.01, hypergeometric). Therefore, we considered the G1/S restriction point CDK inhibitors including p21 (*Cdkn1a*), p27 (*Cdkn1b*), and p57 (*Cdkn1c*), all of which are computationally predicted targets of the *mir-302* cluster^24^. Analysis of expression data from E7.5 through E9.5 showed an upregulation of p27 coinciding with the downregulation of miR-302 (Supplementary Fig. 4H,). To confirm that p27 can be targeted by the mir-302 miRNAs, its 3′UTR was cloned into a reporter construct (Supplementary Fig. 4J). The construct was then transfected into microRNA deficient (*Dgcr8−/−*) and wild-type (wt) ESCs. The reporter showed reduced levels in the wt versus *Dgcr8−/−* cells, consistent with miRNAs regulating their expression. Introduction of a miR-302 mimic, but not a miR-302 seed mutant mimic, into the *Dgcr8−/−* cells re-suppressed the p27 reporter to wild-type cell levels, confirming the miR-302 miRNAs can target p27.

Given that p27 can be targeted by *mir-302* and its expression in vivo is anticorrelated with the miR-302 levels, we next asked whether the loss of p27 would lead to delayed phasic gene transcription, the reciprocal phenotype of the *mir-302−/−* cells. For this analysis, we focused on embryonic day E9.5, when repression of p27 would typically be alleviated due to the downregulation of miR-302 and phasic expression typically established. Differential gene expression analysis between G1 and G2/M showed far fewer genes were phasically expressed in E9.5 *p27−/−* embryos than stage-matched controls (Fig. 2F–I, Supplementary 4K, Supplementary Data 4). This difference was not due to an overall loss of expression of the genes in the mutant embryos (Fig. 2J). *p27−/−* cells did show a slight decrease in G1 cells, although not reaching statistical significance (Supplementary Fig. 4L). Together, these data link regulation of the G1/S restriction point with establishment of phasic expression in the mouse embryo.

### Gain in phasic expression is shared across lineages

Between E7.5 and E9.5, the cellular composition of the developing embryo increasingly diversifies. Expression analysis of whole embryos may not detect genes that show lineage-specific phasic expression and could be confounded by contaminating extra-embryonic and/or postmitotic cells. To address these issues, we transitioned to single-cell RNA-sequencing (scRNA-seq) data. First, using published embryonic single-cell sequencing data we performed a co-expression analysis of canonical G1/S and G2/M genes on mitotic embryonic cells from E6.5 to E8.5^25^, spanning from the pluripotent epiblast through germ layer specification (Supplementary Fig. 5A–C and methods). G2/M genes showed some correlation at E6.5 that increased to strong correlations by E8.5. G1/S genes showed little correlation prior E7.75 that increased to moderate correlations by E8.5. Therefore, consistent with the phase-sorted bulk sequencing data, co-expression analysis of canonical phasic genes from cycling embryonic cells shows a gain in phasic expression over developmental time.

**Fig. 3:**
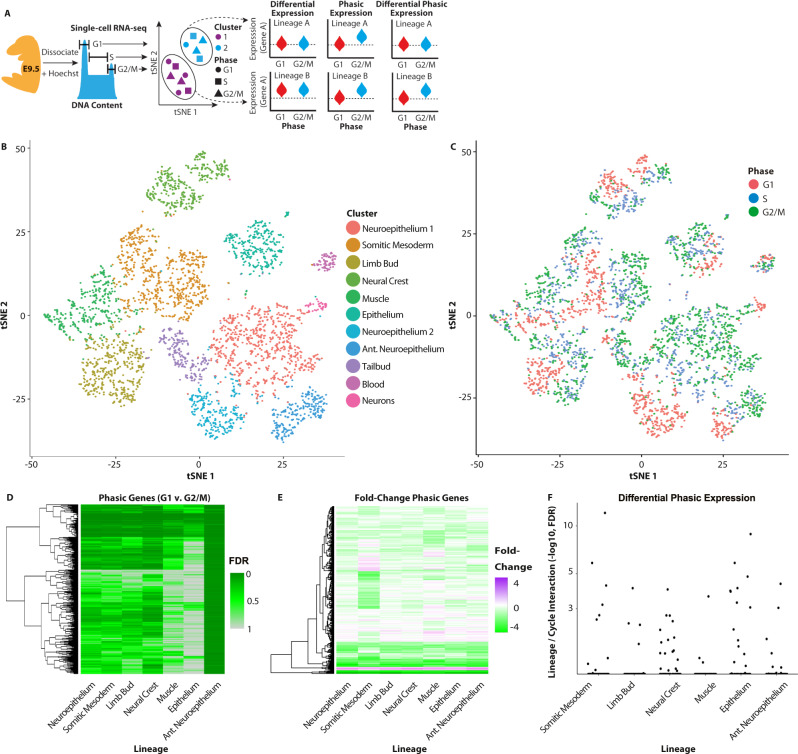
Phasic programs differ between lineages in E9.5 embryos. **A** Schematized workflow for identifying lineage-specific phasic programs. E9.5 embryos were dissociated to single cells, sorted by cell cycle phase based on DNA-content, captured by scRNA-seq, separated into lineages by t-Distributed Stochastic Neighbor Embedding (t-SNE), and then subjected to differential expression analysis accounting for lineage and phase. **B** t-SNE plot of 11 populations identified by shared nearest neighbor analysis of single-cell transcriptomes dissociated from whole E9.5 embryos. 13 PCA dimensions were reduced and depicted in the t-SNE plot. (*n* = 2 biologically independent litters. Litters of 3 and 5 embryos were pooled as input for the respective samples.) **C** The distribution of cells by cell cycle phase as identified by DNA content within the clusters. **D** A heat map of *P* values for all genes within each lineage that showed differential expression between G1 vs. G2/M (BH adj. FDR < 0.05, Hurdle model (see Methods)). **E** The fold-change between G1 and G2/M of genes that are phasically expressed in at least one lineage (adj. *P* < 0.05 in “D”). The source data is provided in “Source Data.xlsx”. **F** The distribution of −log10 FDR values of interaction term between phase and lineage. Dots represent genes that are distinctly phasic in each lineage relative to neuroepithelium (reference lineage) (BH adj. FDR < 0.05, Hurdle model (see Methods)).

Next, we performed de novo discovery of phasic genes that were common and lineage specific by combining sorting of cells in G1 vs. G2/M with scRNA-seq (Fig. 3A, Supplementary Fig. 5D, methods). In two independent collections of wt E9.5 embryos, 574 and 3301 cells with at least 5000 unique molecules per cell were captured (Supplementary Fig. 5E). Comparison of differential expression between cell cycle phases of replicates and bulk RNA sequencing at E9.5 showed good reproducibility (see methods). Analysis of all cells, independent of lineage, showed strong co-expression among G1/S and G2/M canonical cell cycle oscillating genes (Supplementary Fig. 5F). Consistent with the co-expression analysis, de novo identified phasic genes showed a strong enrichment for annotated cell cycle genes (Supplementary Fig. 5G–J, Supplementary Data 5).

To compare phasic expression across cell types, we first assigned the phase-sorted single-cell data to specific populations. The two replicate samples were aligned using Shared Nearest Neighbors and assigned to 11 clusters by their expression profiles (Fig. 3B)^26^. Cells from both replicate samples distributed similarly among the clusters (Supplementary Fig. 5K). Transcript capture efficiency did not appreciably impact the clustering (Supplementary Fig. 5L). Examination of the genes driving the separation of the clusters showed that the clusters represented well-known lineages of the post-gastrulation embryo (Supplementary Fig. 5M). Illustrative markers of the lineages include non-neural epithelium (*Epcam*, *Sox2*); neuroepithelium (*Pax6*, *Sox2*), mesodermal lineages (*Twist*, *Meox1*, and *Pdgfra*), tailbud (*T*), and blood (*Cd34*) (Supplementary Fig. 5M, N). Neuroepithelium was separated into two clusters based on cell cycle phase. As these two clusters expressed common markers and represented reciprocal cell cycle phases, they were considered a single lineage for all downstream analysis. DNA-content analysis showed that cell cycle phase represented a second order of organization that occurred within the clusters (Fig. 3C). Furthermore, unsupervised clustering of cells based on the differential expression of annotated phasic genes between G1/S and G2/M sorted 98% of cells correctly as determined by DNA content, showing that periodic expression is widely established by E9.5 (Supplementary Fig. 5O).

Next, we evaluated differential expression between G1 and G2/M within lineages and compared the result across them using linear models (see methods). To facilitate comparison between lineages, we removed small and/or postmitotic lineages (tailbud, blood and neurons; see cluster sizes in Supplementary Fig. 5P) and down-sampled the remainder to compare an equal number of cells from each phase within each lineage. This analysis identified up to 1233 phasic genes within a lineage (FDR < 0.1) (Fig. 3D, E, Supplementary Data 5). Neuroepithelium was set as the reference lineage. The remaining six adequately sized clusters were then compared to this reference to identify all genes that were differentially phasically expressed (see methods). The analysis identified anywhere from 3 to 25 genes that showed significant differences in phasic expression from the neuroepithelium lineage (Fig. 3F, Supplementary Data 5). The overlap between the two replicate samples confirmed reproducibility of these findings (hypergeometric *P* = 6.65e−04). Furthermore, repetition of the analysis with cells randomly assigned to lineages while maintaining the lineage sizes uncovered no genes with an FDR below 0.88, supporting the robustness of the uncovered differences between lineages. These analyses show that the majority of genes that become phasic by E9.5 are shared across two or more lineages, although a small number are lineage specific.

### miR-302/P27 regulates phasic expression across lineages

**Fig. 4:**
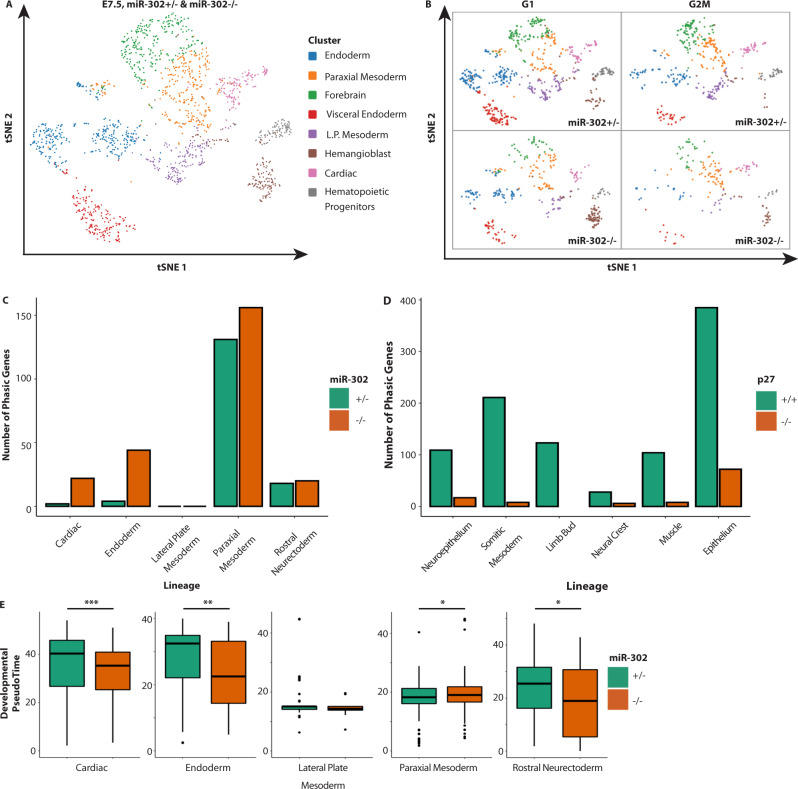
*mir-302* suppresses while p27 promotes phasic expression across multiple lineages. **A** A tSNE plot of 8 populations identified by graph-based clustering of single-cell transcriptomes of cells dissociated from whole control and *mir-302−/−* E7.5 embryos (*n* = 1 litter, 3 *mir-302−/−* embryos and 4 *mir-302*+/− embryos were pooled). **B** The distribution of cells from each genotype and phase within the t-SNE plot. **C** The number of genes showing differential expression between G1 and G2/M (median adj. *P* < 0.1, Hurdle model (see methods)) in each lineage of control and *mir-302−/−* E7.5 embryos. Within each lineage, 100 random samples of the same number of cells from each phase were compared between the genotypes. **D** Same as (**C**), except for control and p27*−/−* E9.5 embryos. **E** The distribution of pseudotime scores for individual cells within each identified lineage of control and *mir-302−/−* E7.5 embryos (Cardiac ***P = 2.07e−5 [*n* = 140], Endoderm **P = 0.0036 [*n* = 315], Lateral Plate Mesoderm *P* = 0.21 [*n* = 178], Paraxial Mesoderm *P = 0.048 [*n* = 293], Rostral Neurectoderm *P = 0.012 [*n* = 267], two-tailed *t* tests, “n” is the number of biologically independent cells of each type). Higher pseudotime score represents further differentiation. See Supplementary Fig. 7A for the basis of the scoring metric. Center, median. Bounds of box, 25th and 75th percentiles. Whiskers, the minima and maxima are the most extreme points within 1.5× the IQR of the box edge. The Source Data is provided in “Source Data.xlsx”.

Next we asked whether the same mir-302/p27 axis uncovered by whole embryo analyses was a common mechanism regulating the onset of phasic expression across lineages. For this analysis, we phase-sorted and profiled single-cell transcriptomes of control and of *mir-302* and *p27* knockout embryos. *mir-302* knockout embryos were profiled at E7.5, when miR-302 transcript levels are highest and only a small number of lineages have been specified. To enable a lineage-by-lineage assessment of the impact of *mir-302* removal on phasic expression we matched cell types in the mutants with those in controls. Mutual nearest neighbors were used to align the *mir-302*-/- cells to the cell types in the control embryos; lineages with more than 50 viable cells were used for downstream analysis (Supplementary Fig. 6A)^27,28^. t-SNE representation uncovered 8 distinct populations at this embryonic stage all represented in the control and knockout embryos with similar proportions (Fig. 4A, B, Supplementary Fig. 6B, C). *mir-302* targets were upregulated in the embryo, but not in extra-embryonic tissue as expected given the miRNA’s highly enriched expression in the embryo (Supplementary Fig. 6D, E)^23^. Differential expression analysis between an equal number of cells in G1 and G2/M in each population showed that *mir-302* loss resulted in a large gain in the number of phasic genes in some lineages (cardiac and endoderm), a smaller gain in others (paraxial mesoderm), and no change in the remainder (lateral plate mesoderm and rostral neuroectoderm) (Fig. 4C, Supplementary Data 6). The differences in these gains likely relate to the varied maturation schedules of the lineages as well as the relative timing of miR-302 decrease in each population as our analysis captured only a single timepoint.

We took a similar approach to evaluate the impact of p27 loss on phasic expression, except we focused on E9.5, when p27 is highest and periodic expression is well established across lineages in wt embryos. We performed a lineage-by-lineage assessment of the impact of p27 removal on phasic expression in embryos using only lineages with at least 30 viable cells for downstream analysis (Supplementary Fig. 6F)^27,28^. Effective alignment of *p27* mutants and controls was supported by concordance of wt lineage marker genes in the aligned p27*−/−* cell types (Supplementary Fig. 6G). Differential expression analysis between G1 and G2/M showed a loss in the number of phasically expressed genes across all the lineages (Fig. 4D, Supplementary Data 6). All together these data show a reciprocal impact of the G1/S restriction point regulators miR-302 and p27 on phasic gene expression across multiple lineages.

### Early phasic expression in mir-302*−/−* precedes differentiation defects

Next we asked how miR-302 mediated suppression of phasic expression relates to its established role in developmental timing of cellular differentiation^22,29^. In particular, given that *mir-302* removal leads to premature differentiation in multiple cell types^22,30^, we asked if the precocious phasic expression seen at E7.5 was upstream or downstream of the differentiation phenotype. To address this question, differentiation and phasic expression were simultaneously assessed in cells of each lineage and compared between the control and *mir-302* knockout embryos. To do this, we established a developmental time scale based on pseudotime analysis of existing E6.5 to E8.5 single-cell sequencing data^25^. Established markers changed as expected along the pseudotime scale (Supplementary Fig. 6H, I and methods). This time scale was then used to determine differentiation status of individual cells from the *mir-302* control and knockout embryos (Fig. 4E). Across lineages, the *mir-302* knockout cells were similar or even slightly behind controls in differentiation. Therefore, the precocious phasic expression seen in the mir-302 knockouts precedes precocious differentiation as defined by pseudotime.

### Phasic transcription factor activity regulates phasic programs

**Fig. 5:**
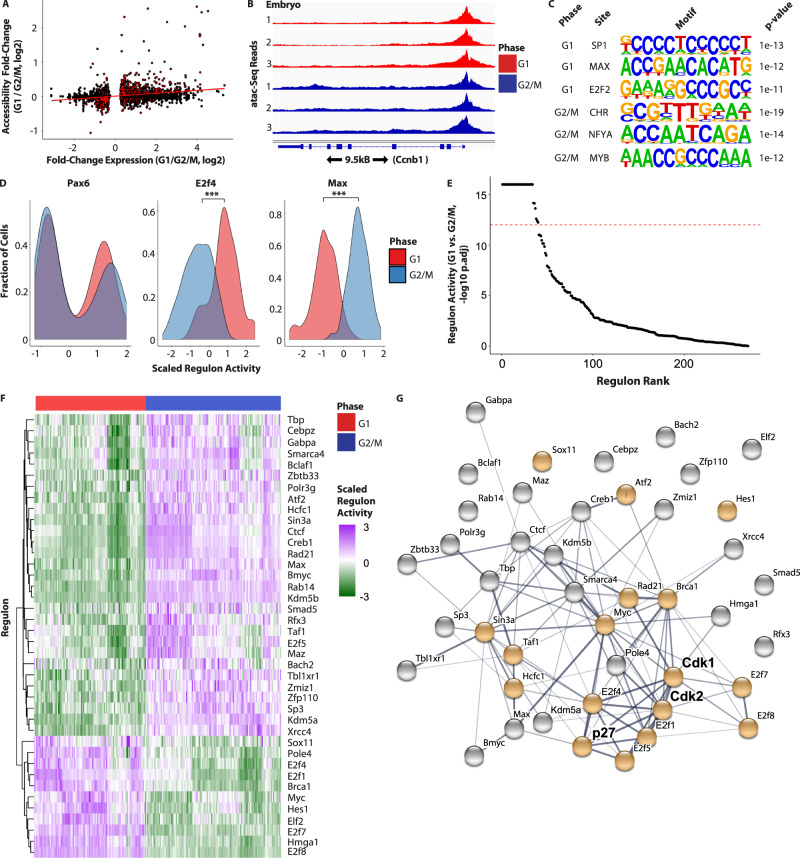
Chromatin accessibility supports the network inferred to regulate phasic expression. **A** Correlation plot of differential accessibility as measured by ATAC-seq and differential expression between G1 and G2/M of E9.5 embryos (Spearman’s rho = 0.24 for all peaks associated with differentially expressed genes and rho = 0.53 for differentially accessible peaks; *P* < 1 × 10e−16 in both cases). Only genes that show differential expression (adj. *P* < 0.05) are shown as red dots. *n* = 3 biologically independent embryos. **B** ATAC-seq tracks of three E9.5 embryos sorted into G1 and G2/M. *CyclinB1* is displayed. **C** Motif enrichment of established regulators of phasic expression within either G1- or G2/M-associated accessible peaks. **D** The distribution of *Pax6*, *E2f4* (***P < 0.001) and *Max* (***P < 0.001) regulon activity by phase evaluated with SCENIC in all E9.5 cells (two-sided Kolmogorov–Smirnov test between G1 and G2/M). **E** The difference in the distribution of regulon activity between cells in G1 and G2/M. All of the regulons inferred by SCENIC among phasically expressed genes are depicted. A two-sided Kolmogorov–Smirnov test was used to evaluate the difference in activity between the phases. The threshold (*P* < 1 × 10^−12^) used to define phase-specific regulons is depicted as a red-dashed line. Source data is provided in “Source Data.xlsx”. **F** The scaled activity of each phase-specific regulon (from **E**) is plotted by row for each E9.5 cell (columns). **G** STRING plot depicting confidence of gene-gene interactions among phase-specific regulons from wt E9.5 embryos based on the STRING database^89^. String-db builds the network based on previously annotated interactions that include co-expression and numerous experimentally determined interactions (e.g., protein–protein interactions). Increasing thickness of connecting lines represents increasing confidence. Included in the STRING plot are p27, CDK2, and all TFs uncovered by SCENIC (see text). Highlighted in gold are genes annotated in gene ontology “Cell Cycle” and “Regulation of the Cell Cycle” categories.

Since neither miR-302 nor p27 directly regulate transcription, we next asked how they might interface with transcriptional networks to drive phasic gene expression. Cells from E9.5 embryos were sorted into G1 and G2/M populations and subjected to ATAC-seq (Supplementary Fig. 7A–C). The nearest accessible chromatin to twenty-eight percent of phasically expressed genes showed a change in accessibility between phases (*P* < 0.01). The change in accessibility and expression of these genes was correlated, although the absolute fold-change was generally small (Fig. 5A, Supplementary Fig. 7D). Importantly, most accessibility peaks associated with phasically expressed genes did not change between phases, as exemplified by Cyclin B1 (*Ccnb1*) (Fig. 5B). Therefore, differential chromatin accessibility does not appear to underlie the majority of differential expression between cell cycle phases.

Next we asked what transcription factors (TFs) could be regulating phasic expression. Motif analysis of accessible regions associated with G1 or G2/M differentially expressed genes uncovered numerous established cell cycle regulators (Supplementary Data 7). For example, the most significantly enriched G2/M motif is the cell cycle genes homology region (CHR) (Fig. 5C). The CHR mediates phasic expression by nucleating DREAM-based repression in G0/G1 and promoting FOXM1-MUVB or MYB-MUVB mediated expression in S/G2/M^31,32^. The MYB motif was also highly enriched in G2/M peaks. Other motifs showing strong enrichment in either the G2/M or G1 enriched set included SP1, MAX, E2F, and NFY. The activities of these TFs is regulated by CDK phosphorylation providing a potential link between restriction point and phasic transcription^33–36^.

To further explore what transcriptional networks were regulating phasic expression, we applied the SCENIC package, which scores for regulon activity based on correlation in expression of a TF and its predicted targets^37,38^. When applied to wt E9.5 single-cell data to identify regulators of the phasically expressed genes, SCENIC identified a list of TFs highly enriched for cell cycle regulators (GO: “Cell Cycle”, hypergeometric *P* = 1.7e−7, Supplementary Fig. 7E). The SCENIC inferred TFs showed a strong overlap with the TFs inferred by motif enrichment analysis described above (hypergeometric *P* = 2.3e−04). The inferred regulons include a mixture of those similarly active in both G1 and G2/M and those preferentially active in one phase (Fig. 5D, Supplementary Fig. 7E). To distinguish TFs that are preferentially functioning in one phase, and thus likely driving phasic expression, we compared the activity of each regulon between G1 and G2/M and selected those showing a strong statistically significant preference for either phase (Fig. 5E–G). The resulting high-confidence relationships included interactions with the canonical G1/S regulators the E2Fs, E2F cofactor *Hcfc1*^39^, classic cell cycle driver *Myc*^36^ and its dimerization partner *Max*^40^, restriction point regulator *Brca1*^41^ and other TFs previously implicated in cell cycle regulation. These analyses suggest that phasic TF activity regulates the onset of phasic gene expression.

### miR-302 suppression of CDKIs regulates differentiation timing

**Fig. 6:**
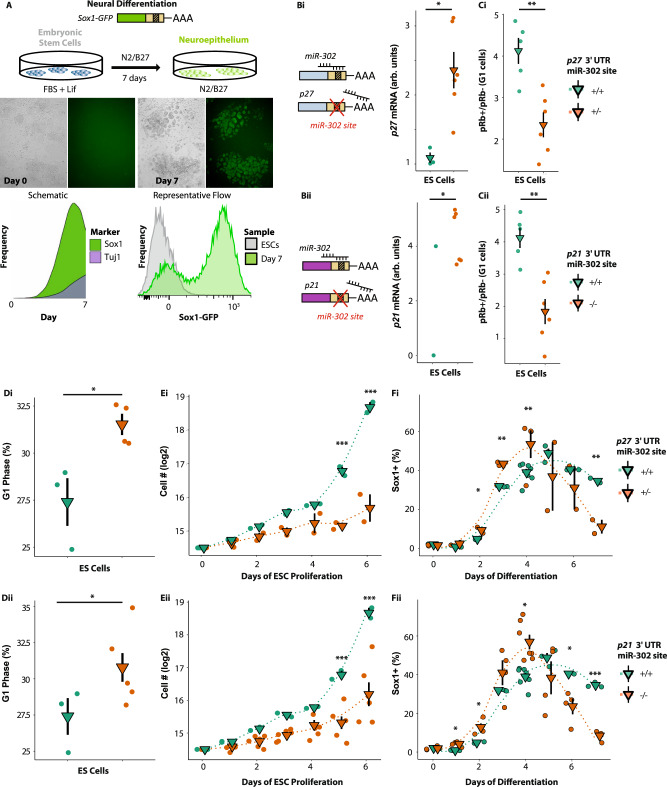
Deletion of miR-302 target sites in p27 and p21 results in precocious differentiation. **A** The differentiation of ESC into neuroepithelium following seeding in N2/B27 is evidenced by a progressive increase in the fraction of cells expressing the neural marker Sox1. This neuroepithelium can undergo further differentiation into Tuj1+ neurons. **B** Deletion of *miR-302* target sites in the 3′UTR of p27 or p21 leads to an increase in their respective transcript abundance in ESCs. QPCR reveals elevated p27 and p21 in the miR-302 binding site mutants (two-tailed *t* tests *P = 0.014 (p27) and *P = 0.025 (p21), 1 control line and 2 biologically independent mutant cell lines in each of three independent experiments). **C**–**E** Deletion of the miR-302 binding site in either p27 (i) or p21 (ii) of ESCs results in (**C**) reduced Rb phosphorylation (two-tailed *t* tests, **P = 0.0027 (p27), **P = 0.0016 (p21), 1 control line and 2 biologically independent mutant cell lines in each of three independent experiments), (**D**) accumulation in the G1 cell cycle phase (two-tailed *t* tests, *P = 0.01 (p27, *n* = 4 (2 biologically independent mutant cell lines and control samples in each of 2 independent experiments)), *P = 0.04 (p21, *n* = 6 biologically independent mutant cell lines and three independent control samples)), (**E**) deficient ESC proliferation (adjusted *t* tests, ***P < 0.001, *n* = 2 (p27) and *n* = 4 (p21) biologically independent mutant cell lines and two independent control samples at each timepoint), as well as (**F**) early neural differentiation (adjusted *t* tests, *P < 0.05, **P < 0.01, ***P < 0.001). The “*n*” at each timepoint is the number of biologically independent mutant cell lines or control samples. Each timepoint is shown as p27/p21/control. *n* = 2/4/2 (day 0), 2/4/3 (day 1), 2/4/3 (day 2), 2/4/3 (day 3), 2/3/4 (day 4), 2/4/3 (day 5), 2/4/3 (day 6), 2/4/3 (day 7). The Source Data for (**C**–**F**) are provided in “Source Data.xlsx”.

To further dissect the link between the G1/S restriction point, phasic expression, and differentiation, we asked if the miR-302 regulation of CDKi impacts ESC differentiation. Previous work showed a link between miR-302 loss and premature neuronal differentiation in vivo^22,42^. Neural differentiation proceeds through Sox1+ neural progenitor cells that can give rise to Tuj1 positive neurons (Fig. 6A). Similar to the in vivo phenotype, *mir-302* knockout ESCs differentiated prematurely down the neural pathway (Supplementary Fig. 8A). Prior to neuronal differentiation, the *mir-302* knockout ESCs showed a premature activation of the restriction point as measured by an increase in hypophosphorylation of the CDK target Rb in G1 at day 3 of differentiation, accumulation in G1 phase of the cell cycle, and a reduced fraction of Cyclin E1+ cells in G1 (Supplementary Fig. 8B–E). Although the number of early neurons in *mir-302* knockouts is modest, a similar cellular phenotype in vivo is upstream of fully penetrant neural tube closure defects and embryonic lethality^22^. To determine if miR-302 was acting directly through the restriction point, we next mutated the miR-302 target sites in the 3′UTR of the endogenous p21 and p27 genes (Supplementary Fig. 8F). The mutants ESCs showed elevated levels of p21 and p27 transcripts respectively (Fig. 6B). They also showed reduced phosphorylated Rb in G1, an increase in the number of cells in G1, and a reduced proliferation rate (Fig. 6C–E, Supplementary Fig. 8G). Upon the induction of neural differentiation, the mutant cells prematurely differentiated exhibiting more Sox1-GFP+ cells than wt early in the differentiation, with the Sox1-GFP+ fraction peaking one day earlier than their wt counterparts, and also declining before wt cells, consistent with further differentiation (Fig. 6F)^43^. Therefore, miR-302 inhibition of the G1/S restriction point not only suppresses premature phasic expression, but also premature cell differentiation, supporting a link between the two.

### Constitutive Cyclin E blocks ESC differentiation

Next, we wanted to know if the establishment of phasic expression promotes differentiation of pluripotent cells. This question was complicated by the large number of genes that become phasic. However, one of these genes, Cyclin E1, is well-known to be phasic in somatic cells, constitutive in ESCs, and to promote progression through the G1/S restriction point. Moreover, Cyclin E1 became phasically expressed between E7.5 and E9.5 (Fig. 1L, Supplementary Fig. 2A). Therefore, we hypothesized that Cyclin E could be an important link between the G1/S restriction point, phasic gene expression, and cellular differentiation.

**Fig. 7:**
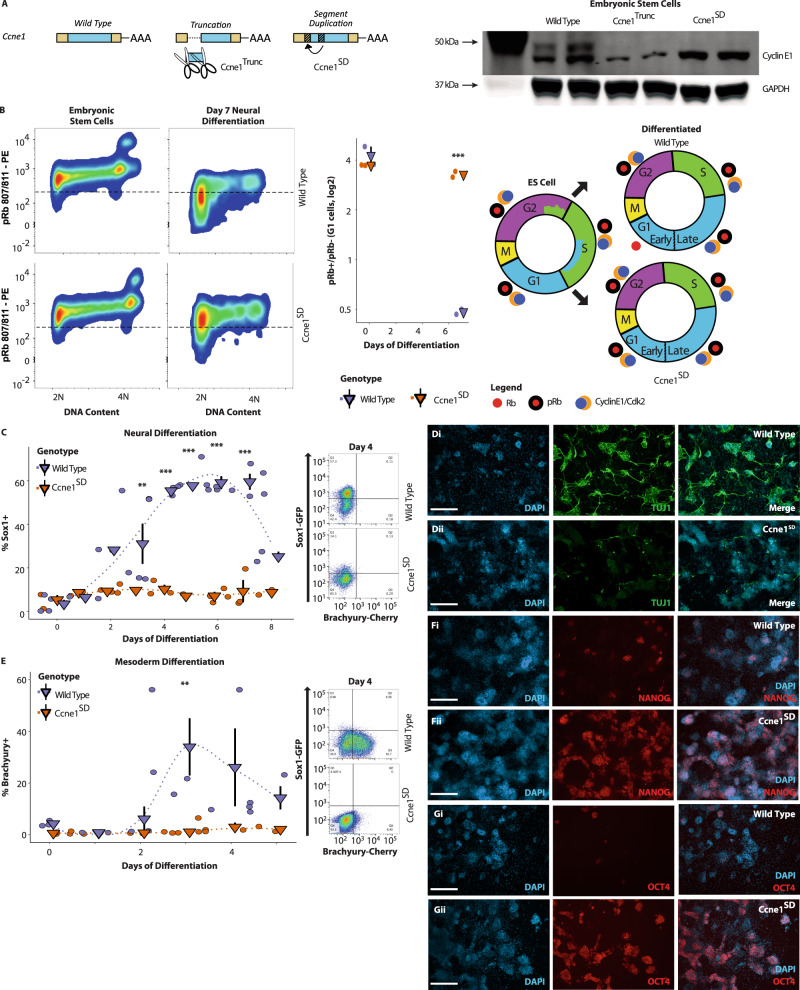
Differentiation is impaired by *Ccne1* mutation promoting a constitutive cell cycle. **A** Targeting *Ccne1* with homology directed repair gave rise to N-terminal truncation and segment duplication (SD) mutants. Cyclin E1 western blot of control, Ccne1^Trunc^ and Ccne1^SD^ mutant ES cell lines. The upper band seen in wt and absent in both mutants is of unknown origin. **B** Rb remains constitutively phosphorylated at site 807/811 of Ccne1^SD^ mutants during assay of neural differentiation. (left, middle panels) The fraction of pRb 807/811+ cells is higher in Ccne1^SD^ than controls after 7 days differentiation (two-way ANOVA, *P* = 0.0031, Sidak’s multiple comparison test ***P = 0.0005 (day 7), *n* = 2 biologically independent samples across two independent experiments). (right panel) Schematic of data from “B” illustrating the presence of pRb throughout G1 in neural differentiation assay of Ccne1^SD^ mutants. **C** Neural differentiation of Ccne1^SD^ mutants is impaired (**P < 0.01, ***P < 0.001, Sidak’s multiple comparison test, two-way ANOVA, *P* < 0.0001). **D** Consistent with deficient neural differentiation, Ccne1^SD^ mutants rarely generate βIII-tubulin+ neurons (scale bar = 200 μm). **E** Mesodermal differentiation of Ccne1^SD^ mutants is impaired (**P = 0.0037, Sidak’s multiple comparison test, two-way ANOVA, *P* = 0.0011). **F**, **G** Rather than neural expression, the expression of pluripotency markers NANOG (**F**) and OCT4 (**G**) persist during neuronal culture of Ccne1^SD^ mutants (scale bar = 200 μm). The Source Data for (**B**, **C**, **E**) are provided in “Source Data.xlsx”.

N-terminal truncation of Cyclin E confers resistance to its phasic inhibition by p21/p27 in some human tumors^44^. Targeting this domain in ESCs to generate a mutant that constitutively bypassed the G1/S restriction point led not only to the intended N-terminal truncation mutant, but also an unintended segment duplication (Ccne1^SD^, Fig. 7A). Unexpectedly, the truncation appeared to destabilize Cyclin E. In contrast, the segment duplication retained wt levels of the protein (Fig. 7A). The segment duplication disrupted exon1 and resulted in a translational start site 18 amino acids downstream of the original start site (Supplementary Fig. 9A). We analyzed the phasic activity of these mutants by evaluating Rb phosphorylation (pRb) across the cell cycle during neural directed differentiation. Both wild-type and mutant cell lines showed constitutive Rb phosphorylation in ESCs. However, while wt and the truncated mutant cell lines showed a gain in phasic Rb phosphorylation during ESC differentiation (Supplementary Fig. 9B), the segment duplicated mutant line retained constitutive Rb phosphorylation even after 7 days in neural differentiation conditions (Fig. 7B). Furthermore, while Cyclin E1 protein levels in G1 became varied in wt cells, they remained consistent in the Ccne1^SD^ cells after 4 days neural differentiation (Supplementary Fig. 9C).

The different mutants were made in a Sox1-GFP, Brachyury-RFP dual reporter ESC background allowing real-time visualization of each marker throughout a differentiation time course^45^. Neural directed differentiation of wt ESCs and the truncated mutant (Cnce1^Trunc^) cells showed a progressive increase in Sox1 reporter expression, peaking after 6 days of differentiation with ~60% of the cells being Sox1-GFP+ (Supplementary Fig. 9D, E). In contrast, Ccne1^SD^ mutant cells failed to upregulate Sox1 at any point throughout the time course (Fig. 7C, Supplementary Fig. 9D). Similarly, staining for Tuj1 at day 10 of differentiation showed a large number of Tuj1 positive wt cells, but very few Tuj1+ Ccne1^SD^ mutant cells (Fig. 7D). Neural differentiation of Ccne1^SD^ mutants was rescued by CDK2 inhibition, ruling out a secondary mutation underlying the differentiation defect (Supplementary Fig. 9F). Furthermore, these differences could not be explained by apoptosis as Ccne1^SD^ mutant cells showed reduced staining for the apoptotic marker Annexin V (Supplementary Fig. 9G). ESC proliferation of Ccne1^SD^ mutant cells was unchanged (Supplementary Fig. 9H). In contrast, the number of Ccne1^SD^ mutant cells and the percent of cells in S phase was higher following 7 days in neural differentiation conditions, consistent with bypass of the G1/S restriction point and increased proliferation (Supplementary Fig. 9I, J). To validate with an alternative system, we developed a doxycycline inducible CRISPR activation (idCas9-VPR) cell line that is expected to result in constitutive expression (Supplementary Fig. 9K). Introduction of a guide RNA to the *Ccne1* promoter resulted in a 25% increase in Ccne1 mRNA levels even in the absence of doxycycline and 50% increase with the addition of doxycycline (Supplementary Fig. 9L). When the idCas9-VPR cells were placed under neural differentiation culture conditions, there was a negative correlation between the increase in Ccne1 and percent Sox1-GFP+ cells after 7 days, again consistent with non-phasic Cyclin E inhibiting ESC differentiation (Supplementary Fig. 9M).

Next, to evaluate whether constitutive Cyclin E specifically blocked neural differentiation, we tested mesodermal differentiation of the mutants. While wt and Cnce1^Trunc^ cells showed a maximal fraction of Brachyury reporter positive cells at day 3 of differentiation, Ccne1^SD^ cells failed to induce reporter expression at any timepoint (Fig. 7E, Supplementary Fig. 9N, O). To determine if the failure to turn on these markers was secondary to a general block in differentiation or a skewing to alternative lineages, we stained for the ESC markers NANOG and OCT4 after directed neural differentiation for 10 days and found their expression was mutually exclusive with neural and neuronal markers (Supplementary Fig. 9O, P). Strikingly, while wt cells showed an almost complete downregulation of these markers, the Ccne1^SD^ cells retained robust levels of both pluripotency proteins (Fig. 7F, G). To assess how Ccne1^SD^ impacted differentiation we compared acquisition of phasic expression between mutants and controls. Consistent with Ccne1^SD^ impairing differentiation, mesodermal markers were lower in Ccne1^SD^ cells than controls following 4 days in mesodermal differentiation conditions (Supplementary Fig. 10A–C). Furthermore, as expected, fewer genes became phasic in Ccne1^SD^ cells (Supplementary Fig. 10D–I, Supplementary Data 8). Together, these data show that the transition from constitutive to phasic Cyclin E promotes phasic gene expression and ESC differentiation.

## Discussion

Our findings support a model where G1/S restriction point establishment during early embryonic development and ESC differentiation results in the onset of phasic expression of many genes and promotes differentiation. The repression of G1/S restriction point components, including the CDK inhibitors, is at least in part driven by microRNAs from the miR-302 and miR-290 clusters, which share a common seed and are highly enriched in ESCs^46^. During differentiation, expression of miR-302 rises as miR-290 falls, before miR-302 itself is shut-off between E7.5 and E9.5. In *mir-302* knockouts, precocious phasic expression is gained at E7.5 preceding premature neuronal differentiation, defective neural tube closure and embryonic lethality^22^. Derepression of p21 and p27, by deletion of the miR-302 target sites in their 3′UTR, similarly results in premature differentiation, with the peak fraction and the decrease of the Sox1+ cells occurring approximately one day earlier than normal. In addition to miR-302a-d, the miR-302 cluster contains miR-367, which is predicted to target p57. Therefore, the miR-302 cluster is likely to temporarily inhibit all three CDKis in early mouse development thereby suppressing the restriction point, phasic expression, and premature differentiation. Deletion of p27 alone causes differentiation defects in the thymus^47^, pituitary^47^, and adrenal glands^47^ as well as the ovaries^47^, testes^47^, spleen^47^, retina^47^, cochlea^48,49^, neurons^50^, and beta-cells^51^. Moreover, p27 is partially redundant with p21 and p57, and while a triple CDKi knockout has not been made, the loss of both p21 and p27 alleles, and p57 allele results in embryonic lethality around E13.5 secondary to a combination of placental defects and various embryonically skeletal and gastrointestinal abnormalities associated with increased apoptosis and abnormal differentiation^52–54^. Hence either the absence or premature induction of CDKis disrupts development. How defects in the G1/S restriction point cause developmental timing abnormalities remains only partially understood.

The G1/S restriction point links phasic expression and differentiation but the extent to which these effects occur in series or in parallel remains unknown. More granularly, whether and how the phasic nature of each gene is integral to the differentiation of the cell types in which they become phasic at E9.5 is unclear. We demonstrate that the phasic nature of at least one of the genes that transitions from constitutive to phasic expression through the cell cycle, Cyclin E, is essential for ESC differentiation. Cyclin E itself regulates the G1/S transition by associating with CDK2 and phosphorylating targets to promote G1 to S transit including Rb. Therefore, the establishment of a G1/S restriction point through the derepression of the CDK inhibitors appears to initiate a temporal window where CDK2 is inactive that is required for differentiation. How cell cycle regulated changes in the abundance of other phasic genes across the cell cycle contribute to development is an exciting future direction.

Understanding how phasic expression and differentiation cues interact will provide fundamental insights into the molecular mechanisms underlying cell fate transitions. Recent studies provide insights into such mechanisms. In particular, Cyclin D1-3 modulates the response of TGF-beta signaling in human ESCs resulting in distinct cell fate choice depending on the timing of signal within the cell cycle^15^. Also, the fusion of trophoblast cells into a syncytium is regulated by the interaction of the phasic gene p21 with the tissue specific TF *Gcm1* restricting *Syncytin-2* transcription to G0/G1 phase of the cell cycle^55^. We expect that many other examples of direct links between cycle machinery and tissue specific differentiation programs are yet to be discovered.

## Methods

### Cell culture

Mouse ESCs were grown in 15% FBS (Corning) and LIF. Most ESC experiments were performed in Sox1-GFP, Brachyury-RFP dual reporter cells (a kind gift from the Suter lab)^45^. The exceptions were *mir-302*−/− experiments, where mutants from ref. ^22^ were used along with their V6.5 parental controls. 3T3 cells were grown in 10% FBS (Corning).

### Mouse husbandry

All mice were maintained in accordance with the UCSF animal husbandry guidelines. In brief, mice were maintained on a 12 h light/dark cycle, and housed in standard temperature and humidity controlled rooms. Mice were bred as trios and housed 5 per cage otherwise. All experiments were reviewed and approved by the UCSF Animal Care and Use Committee. The *mir-302*−/− and *p27*−/− embryos were genotyped as their respective founders were^22,56^. All mice were maintained by mating to C57Bl/6J females (Jackson Laboratory). Embryos were staged and pooled according to morphology:^57^ primitive-streak stage embryos reported as E6.5 days post coitum (d.p.c.), head-fold stage embryos were reported as E7.5, embryos with 1–15 somites reported as E8.5 and embryos with 20-35 somites reported as E9.5. To compare phasic expression between individual mutant and control embryos (GFP+ mir-302−/− at E8.5 and p27−/− at E9.5), cohorts were matched within 2 somites.

### Embryo dissociation

Embryos were dissociated at 37 °C in HBSS containing 1% trypsin and 10 μM EDTA following removal of extra-embryonic tissue. Dissociation lengths were stage-matched: E6.5 for 5 min, E7.5 for 7 min, E8.5 for 10 min, and E9.5 for 15 min. In each case, gentle agitation with a 1 ml pipette was used to aid dissociation. Dissociated cells were resuspended in HBSS + 2% FBS and filtered through 0.45um cell strainers (Fisher Scientific). Every sample reported in this study is independent in that it is derived from distinct embryos and never measured repeatedly.

### DNA-content analysis

For whole embryo DNA-content profiles (Fig. 1), embryos dissociated to single cells were strained through a 40 μm cell strainer, resuspended in 500 μl of PBS and fixed in 5 ml of 70% ethanol for 2 h on ice. The cells were intermittently vortexed to prevent aggregation. These fixed cells were rinsed twice in PBS, and DNA stained with FxCycle (Propidium Iodide/RNase, ThermoFisher, F10347). DNA-content profiles were captured on an LSRII. For all RNA-seq experiments, live cells were dissociated as described, and stained for DNA content by incubation with 10 ng/ml Hoechst 33342 (ThermoFisher) in HBSS + 2% FBS for 40 min at 37 °C prior to sorting.

### Flow cytometry and FAC-sorting

All sorting was performed on a FACS Aria2 using FACSDiva v8.0.1. Cells were sorted for DNA-content using Hoechst 33342 as illustrated in Supplementary Fig. 2A–C. Analysis of flow data was performed using FloJo v10.8.1.

### Bulk RNA-seq

Sorted populations were resuspended in Trizol LS (ThermoFisher) and RNA isolated according to the manufacturer’s instructions. 3′ end capture libraries were prepared using Quant-seq (Lexogen) and full-length libraries using Kapa RNA Hyper-prep kits (Kapa Biosystems), per manufacturer’s instructions. Single-end 50 bp sequencing of these libraries was then performed on a HiSeq4000.

### miRNA-seq

miRNA libraries were prepared as described in ref. ^58^. In brief, miRNAs were purified from total RNA using the miRNeasy kit (Qiagen). miRNAs were then ligated to 3′ adaptors with NNNN at each 5’ end to reduce ligation bias and a unique barcode for each sample. Each 3′ ligated sample was then size-selected for miRNAs and pooled. The pool was ligated to a common 5′ adapter, reverse-transcribed, amplified for 14 cycles and single-end sequenced on a Hi-seq4000.

### Immunohistochemistry

Embryos were fixed in 4% PFA, mounted in 30% sucrose, and 10 μm sections were stained using a Chicken anti-GFP primary antibody (1:200, Aves Labs, GFP-1010) and 488-Donkey anti-Chicken secondary antibody (1:400, Jackson, 703-546-155).

### p27 reporter

A 216 bp region of the p27 3′ UTR encompassing the miR-302 target site was cloned into the pBUTR plasmid to report the regulation of p27 by miR-302^59^. In brief, the region of interest was amplified using GGGGACCCAGCTTTCTTGTACAAAGTGGTCTTCGACGCCAGACGTAAAC and GGGGACAACTTTGTATAGAAAA GTTGGGTGTCCAATGCTTTTAGAGGCAGA using cDNA from whole mouse embryos as a template. This amplicon was then gateway recombineered into the pBUTR plasmid^59^. The reporter was then co-transfected with pCMV-piggybac into wild-type V6.5 and *Dgcr8−/*− ESCs^60^. Cells with integrated reporter were FAC-sorted by GFP. 25 μM of miR-302b-3p and seed mutant mimics (Dharmacon) were transfected into the ES lines using Dharmafect1 (Dharmacon) to assess their impact on translation of the p27 reporter.

### Single-cell transcriptome capture

Single-cell transcriptomes of all E9.5 embryos were captured using Drop-seq^61^, with a few revisions to the published protocol: (1) we added 0.5 M NaCl to the lysis buffer to enhance RNA binding, (2) used beads manufactured by Biosearch (custom), (3) used a flow-rate of 3 ml/h for both the cell suspension and beads as well as a flow-rate of 11 ml/h for the oil, (4) a custom fabricated PDMS device that generates droplets 120um in diameter, and (5) used 6000 beads per PCR reaction. 100,000 cells were inputted from each phase of each of the two E9.5 samples. Viability of all input populations exceeded 90%. Cells were resuspended at 100,000 cells per ml, with beads at 120,000 beads per ml to target 5% cell capture. All beads were RT-PCRed and paired-end sequenced (21 × 75 bp) on a HiSeq4000. The E7.5 *mir-302−/*− and control embryos were captured using Chromium V2 (10X Genomics) per manufacturer’s instructions, and paired-end sequenced (25 × 75 bp) on a HiSeq4000.

### Drop-seq device fabrication

Photoresist masters were created by spinning on a layer of photoresist SU-8 (Microchem) onto a 3 inch silicon wafer (University Wafer), then baked at 95 °C for 20 min. The photoresist masters were then subjected to 3 min ultraviolet exposure over photolithography Drop-seq masks (CAD/Art Services) printed at 12,000 DPI. After ultraviolet exposure, the wafers were baked at 95 °C for 10 min and then developed in fresh propylene glycol monomethyl ether acetate (Sigma Aldrich) before being rinsed with fresh propylene glycol monomethyl ether acetate and baked at 95 °C for 5 min to remove solvent. The microfluidic devices were fabricated by curing poly(dimethylsiloxane) (10:1 polymer-to-cross-linker ratio) over the photoresist master. The devices were cured in an 80 °C oven for 1 h, extracted with a scalpel, and inlet ports added using a 0.75 mm biopsy core (World Precision Instruments, cat# 504529). The devices were bonded to a glass slide using O_2_ plasma treatment and channels that were treated with Aquapel (PPG Industries) to render them hydrophobic. Finally, the devices were baked at 65 °C for 20 min to dry the Aquapel before they were used.

### Phasic expression analysis of Bulk RNA-seq

Genes that did not exceed 5 CPM across one set of replicates (i.e., 2 E7.5 G1 samples) were not included in the analysis. Quality control of samples was performed using Uniform Manifold Approximation and Projection (UMAP) on global expression. All 3T3 and ESC samples were included. Embryonic samples that did not cluster with replicates in UMAP plots were excluded from the analysis, and the dataset rebalanced to compare 2 replicates at each stage (Supplementary Fig. 2D). Inspection of the expression profiles suggests that the failure of one sample to cluster with replicates was likely due to contaminating yolk sac. The remaining samples did not show evidence of significant extra-embryonic contamination (Supplementary Fig. 2E). The filtered dataset was then normalized using the trimmed mean of m-values method^62^. Differential expression was evaluated with a Wald Chi-squared test with a Benjamini-Hochberg correction in DESeq2 v1.22.2 in R v3.5.1 on genes with average expression greater than 5 CPM^63^. Significant differential expression was assessed at adjusted *P* values < 0.1. To evaluate the relative contribution of differential stability and transcription across the cell cycle to phasic gene expression we used limma v3.40.6 in R v3.6.0^64,65^. Phasic expression was evaluated by comparing exonic abundance between G1 and G2/M using the Empirical Bayes method^64,65^. Phasic transcription was evaluated in the same manner as expression, except using intronic data as the input. Finally, differential stability was evaluated as described by ref. ^19^ as the difference between phasic expression and phasic transcription. GSEA analysis was run using enrichR v2.1 in R v3.5.1^66^. Mouse orthologs of human Cyclebase genes were obtained using bioMart v2.50.3^18,67^.

### miRNA expression analysis

Reads were trimmed and demultiplexed using Cutadapt v1.18^68^. mIRNA sequences were aligned to mature mouse miRNAs in miRBase v21 using STAR v2.7.1a with default settings^69^. Only uniquely aligned reads were retained. Aligned reads of 20–24 bp were counted and normalized to counts per million before differential expression was assessed using a moderated t-statistic in limma v3.38.3^64^ in R v3.5.1. Here the Benjamini-Hochberg method was used to calibrate the false-discovery rate associated with the test number. For visualization, miRNAs were grouped into families based on their annotation in Targetscan.

### Single-cell transcriptome analysis

Reads from Drop-seq libraries were processed into a cell by gene count matrix using the Drop-seq v1.12 pipeline^61^ with the following adjustments. The number of contiguous A’s in the polyA trimmer was increased to 12. Reads were aligned to mm10 and Ensembl GRCm38 transcripts using STAR v2.7.1a^69^, and multi-mappers discarded during alignment. Only cells exceeding a threshold of 5000 unique molecules and below a threshold of 5% mitochondrial reads were analyzed. To ensure “spread of signal” was not confounding our analysis, we looked for presence of the same cell barcode associated with multiple indices and found only eight effected cells^70^.

#### Lineage assignment

Cells captured by Drop-seq were parsed into lineages using the default settings of Seurat2^26^ with the following adjustments: (1) cells with fewer than 5000 UMI were filtered from the matrix, (2) the minimum number of genes per cell was increased to 2250 and up to 10,000 were accepted, and (3) the top 15 principal components to identify clusters. The two replicates of E9.5 data were aligned using canonical correlation analysis^71^. The p27−/− data was aligned to the E9.5 reference by mutual nearest neighbors^27,28^. The E7.5 cells captured by Chromium (10X Genomics) were parsed into lineages using default settings of the Cell Ranger v3.0.2 pipeline and visualized by LOUPE v3.0.1^72^. E7.5 embryonic cells with fewer than 5000 UMI were also filtered, and contaminating blood cells were also removed.

#### Pseudobulk

Pseudobulk analysis was used normalize for the high drop-out rate and variable signal associated when analyzing single genes in single cells. Given that the transcript capture efficiency in Drop-seq is estimated to be ~10%^7^, we summed the UMIs for each gene from 10 cells of the same cell cycle phase and lineage. For the permutation analysis, we randomized the cohorts of 10 cells comprising each cell of pseudobulk. To assess whether the interaction between cell cycle phases was specific to particular lineages we used randomized pseudobulk. For this empirical negative control, we scrambled the lineage to which each cell was assigned such that each cell of pseudobulk was from the same cell cycle phase but from mixed lineages. To avoid discrepancies associated with differences in statistical power, we maintained the same number of pseudobulk cells in both the actual and scrambled pseudobulk.

### Phasic expression analysis of single-cell RNA-seq

#### Expression correlation of phasic genes in unsorted populations

A Pearson correlation in the expression of classic cell cycle regulated genes was used to evaluate the degree of their co-expression. Cells from a mouse atlas spanning the pluripotent epiblast to early organogenesis were separated by stage and lineage germ layer using the author’s annotation^25^. Then expression correlation was evaluated in 50 cell cohorts drawn at random without replacement from each stage, within each germ layer. Cell cycle regulated genes were taken from ref. ^9^ focusing on the G1/S (*Ccne1*, *Cdc6*, *Cdc25a*, *Chaf1b*, *E2f1*, *Mcm5*, *Mcm6*, *Pcna* and *Slbp*) and G2/M (*Aurka*, *Birc5*, *Ccna2*, *Ccnb1*, *Ccnf*, *Cdc20*, *Cdk1*, *Cenpa*, *Cenpe*, *Cenpf*, *Cks2*, *Plk1, Racgap1*, *Top2a*, and *Ube2c*) transitions.

#### Between cells classified into phases by FACS

As during lineage assignment, we required a minimum of 5000 UMI per cell for inclusion in the analysis. Further, we required that cells express at least 40% of the genes expressed in the cell with the most detected genes for inclusion in the analysis. At the gene-level, we filtered the matrix of genes that were expressed in <20% of all cells. To call differential expression between phases, we adapted an implementation of the Hurdle model within Model-based Analysis of Single-cell Transcriptomics (MAST) v1.8.2 in R v3.5.1^10,73^. It achieves enhanced sensitivity by considering both the frequency with which a gene is expressed in the population and the expression values within cells where it is expressed^10^. To identify phasic expression in all cells or in subsetted lineages, we used MAST to fit the Hurdle model for each gene in the dataset^73^. The Hurdle model combines two tests. The first is a discrete measure to compare the fraction of cells in each cohort where each gene was detected, while the second is a continuous measure that compares the abundance of each gene amongst cells in the cohort where expression is detectable. To globally assess differential expression between phases, we applied the model using cell cycle phase as a biological covariate and the number of expressed genes as a technical covariate to approximate RNA capture efficiency^10^. To account for the technical variable of transcript capture efficiency per cell, we included the fraction of genes expressed per cell relative to the cell with the most detected genes and tested the significance of cell cycle phases as a parameter.

$$\sim cell\_cycle\_phase\,+\,fraction\_of\_detected\_genes$$

Further, we required an absolute fold-change of greater than 1.1 on the log2 transformed data. Given the low capture efficiency of single-cell RNA-seq, we combined the read counts of ten cells producing “pseudobulk” samples for each phase in order to increase sensitivity for calling differential expression between phases. Since no combination of ten cells recapitulates the actual composition of an individual cell, we generated and analyzed one hundred permutations of the pseudobulk, then took the median false-discovery rate (FDR). Applying a model considering only cell cycle phase and capture efficiency, detected 355 and 1724 genes that were differentially expressed between G1 and G2/M in each of the two samples. The smaller number in the first sample was expected given its smaller sampling size. 246 genes were genes differentially expressed between G1 and G2/M in both datasets; this extensive overlap between the two independent collections (hypergeometric *P* = 6.71e−121, FDR < 0.05), supports reproducibility of the approach.

Similar to bulk analyses, a majority of genes, such as *Hprt*, were not differentially expressed. A total of 1833 phasic genes were uncovered between the two samples including canonical periodic genes such as the G1 cyclin *Ccne2* and the kinetochore protein *Cenpf* (Supplementary Fig. 5G–I). Importantly, the genes identified by this single-cell pseudobulk approach and the regular RNA bulk-seq significantly overlapped (*P* = 9.18e−16, hypergeometric test). Overall gene ontology analysis of the differentially expressed genes showed a strong enrichment for “Cell Cycle” Reactome Pathway (adjusted *P* = 7.66e−45, Supplementary Fig. 5J). Furthermore, the phasic genes were most enriched as a target set of “Encode Transcription Factors (TF)” E2F4 and FOXM1 that are established cell cycle regulons (adjusted *P* = 2.95e−69 and 3.53e−46)^74,75^. These analyses confirmed the value of the single-cell sequencing data in calling phasically expressed genes.

To compare phasic expression between mutants and control embryos, we parsed the embryos into lineages based on their cluster assignment, randomly sampled an equal number of cells from each phase of the control and mutant embryos, and then applied MAST to identify differential expression as described above^73^. Phasic expression of each gene was evaluated as the median *P* value, adjusted for multiple tests using the Benjamini-Hochberg method, from 100 permutations of this comparison. GSEA of the genes that were either precocious or delayed in their periodic expression was performed with the R implementation of enrichR^66^.

### Lineage-specific periodic expression

To identify differential expression that differed between lineages, we utilized the lineages assigned from Seurat2^71^ and extended the linear Hurdle model to include both phase and lineage. To ask if phasic expression was significantly different in any lineage than in a reference lineage, we added a lineage covariate to the linear model and assessed whether significant interactions between lineage and cell cycle phase occurred.

$$\sim cell\_cycle\_phase\,+\,lineage\,+\,cell\_cycle\_phase:lineage\,+\,fraction\_of\_detected\_genes$$

To determine whether the variance inherent in capturing cellular transcriptomes would stochastically create false interactions between periodic expression and lineages, we reran the analysis with scrambled lineage identities. Rather than aggregating 10 cells from the same lineage, we aggregated 10 cells from the same cell cycle phase but mixed lineages. We reasoned that if the observed differential periodic expression was partially or entirely due to noise it would also be found in this lineage-permuted data; however if it was biologically driven, it would be more prevalent when pseudobulk with matched lineages was analyzed. Differential periodic expression related to lineages was detected exclusively on matched-lineage pseudobulk.

### Evaluation of differentiation status in single cells

We aligned the transcriptomes of cells to a reference atlas (E6.5-E8.5) encompassing the E7.5 *mir-302*-/- embryos in developmental time and used pseudotime values as a metric for the maturity of individual cells^25,76^. Since current methods of ordering cells in pseudotime are unreliable for datasets containing many lineage branch points, we first subsetted the atlas by germ layer. Then, for each germ layer, we used the Seurat3 implementation of mutual nearest neighbors to align the *mir-302*± and *mir-302*−/− transcriptomes to the E6.5-E8.5 atlas^27,77^. A distribution of markers recapitulating known differentiation events for each germ layer was confirmed by visual inspection of UMAP plots. The aligned UMAP coordinates were then imported into Monocle3 and pseudotime was evaluated using automated root calls^76^.

### Assay of transposase accessible chromatin in E9.5 embryos

Three E9.5 embryos were phase-sorted by DNA content and 50,000 viable cells were collected from G1 and G2/M to assay transposase accessible chromatin (ATAC-seq). ATAC-seq libraries were prepared by standard methods^78^. Each cohort of cells was tagmented for 30 min and amplified for a total of 9 PCR cycles. Bioanalyzer traces were run to ensure nucleosome peaks were evident in each sample prior to single-end 50 bp sequencing on the HiSeq4000.

### Analysis of chromatin accessibility

Quality control of the samples was performed with ChIPseeker v1.32^79^. To identify peaks of differential chromatin accessibility, the libraries were processed with the following workflow. TN5 sequences were trimmed using cutadapt^68^, and these sequences aligned to GRCm38 using bowtie2 with the following parameters:–local–very-sensitive-local–no-unal–no-mixed–no-discordant–phred33-I 10-X 2000^80^. Mitochondrial reads were then removed using Samtools v1.9^81^, and duplicates removed using Picard Tools v2.20.2 (Broad Institute). Peaks were then called on paired reads with an adjusted *P* value threshold of *q* < 0.1 using Macs2^82^. Peaks were then intersected using Bedtools v2.27.1^83^, and peaks with differential accessibility called using a log2 fold-change threshold of >0.5 and FDR < 0.1 (Benjamini-Hochberg method) using limma v3.38.3^64^. Finally, peaks were annotated using Homer v4.9.1^84^.

### Regulon inference

Regulons were inferred using the SCENIC v 1.1.2-2 package^37,38^. SCENIC was run using default parameters with the exception that GENIE3 v1.4.0 was used to build the co-expression network using only genes that were differentially expressed between G1 and G2/M by MAST (FDR < 0.1).

### Cell line mutation

#### mir-302 target site deletion in Cdkn1b (p27) and Cdkn1a (p21)

Two single guide RNAs (sgRNAs) encompassing the miR-302 target site in the 3′ UTR of *Cdkn1a* and *Cdkn1b* were designed using the Broad Institute’s sgRNA designer. A pair of sgRNAs upstream and downstream of each target site were cloned into the px458 plasmid with either a GFP or BFP reporter to enable sorting of co-transfected cells. To target the 3′ UTR of *Cdkn1b*, we used the guide sequences: ATATCGCTGACTCCATTGAA, CCAATGCTTTTAGAGGCAGA, CATCACTGCTTTATGAAGCA, and GCTACATCCAATGCTTTTAG. To target the 3′ UTR of *Cdkn1a*, we used AGCACTTTGGAAAAATGAGT, CAAAGTGCTATTCAGGTCTG, CAGGTCTGAGGATCACCCCC, and CTCAGACCTGAATAGCACTT. A pair of px458 plasmids targeting up- and downstream of each miR-302 target site was co-transfected into SBR ESCs using Fugene6 (Promega)^45^. 72 h after transfection, cells positive for both GFP and BFP were FAC-sorted, plated at clonal density and genotyped.

#### Cyclin E1 mutation

To truncate *Ccne1*, we used CRISPR-Cas9-enhanced homology directed repair (HDR)^85^. To generate the repair template, we amplified targeting arms using primers 5′ (caggagcagccggctcgacagccagc [F], catggctcaggacttgggct [R]) and 3’ (gaagaaattgccaagattgacaagactg [F], ctatagtccccagccattgctggaggcaaagag [R]) to the intended truncation, and then then fused these arms into an HDR template by nested PCR with the terminal primers. sgRNAs targeting the termini of the intended truncation were then cloned into either px458-BFP or px458-GFP to enable sorting of co-transfected cells (CAAGTCCTGAGCCATGCCAA, TCTTGGCAATTTCTTCATCT)^86^. The two px458 Cas9 plasmids were then co-electroporated (Amaxa nucleofector) with the targeting construct into SBR cells^45^. GFP/BFP double-positive cells were then FAC-sorted, plated at clonal density and genotyped.

### Quantitative RT-PCR

cDNA was prepared using the Maxima RT kit (ThermoFisher), relative transcript abundance evaluated using PerfeCTa SYBR green FastMix Rox (QuantaBio). The primer pairs used to amplify each transcript were taken from PrimerBank^87^: Gapdh (AGGTCGGTGTGAACGGATTTG, GGGGTCGTTGATGGCAACA), Rpl7 (CTGCTGGGCCAAAAACTCTCA, CCTTCAACTCTGCGAAATTCCTT), Cdkn1a (CCTGGTGATGTCCGACCTG, CCATGAGCGCATCGCAATC), Cdkn1b (TCAAACGTGAGAGTGTCTAACG, CCGGGCCGAAGAGATTTCTG), Ccne1 (CTCCGACCTTTCAGTCCGC, CACAGTCTTGTCAATCTTGGCA).

### Neural and mesodermal differentiation

Both the neural and mesodermal differentiation were based on the protocol of ref. ^88^. In brief, for neural differentiation 100,000 dissociated ESCs were seeded in each well of a 6-well gelatin-coated plate in N2/B27. Media was replaced daily over the course of the differentiation. For neuronal differentiation, cells 7 days into the neural differentiation assay were dissociated and reseeded onto Fibronectin/poly-D-Lysine and cultured in N2/B27. Plates were coated in 1:100 Fibronectin (Sigma, F1141) and 50% poly-D-Lysine (Gibco, A3890401) diluted in PBS for 1 h at room temperature prior to seeding. 25 μM of CDK2 inhibitor SC 221409 (ab145053, Abcam) was used during neural differentiation.

For mesodermal differentiation 300 000 dissociated ESCs were seeded in each well of a 6-well gelatin-coated plate in N2/B27. Media was replaced daily over the course of the differentiation and 3uM CHIR99021 (Peprotech, 2520691) was added to the culture after 2 days for the duration of the differentiation.

### Immunostaining

#### Western blots

RIPA lysates containing protease inhibitor cocktails (Sigma, P8340) were run on 10% PAGE gels and transferred overnight. 1:1000 of the primary Cyclin E1 antibody (Cell Signaling, #20808), and 1:10,000 of TUBULIN (ThermoFisher, #236-10501) were used for target detection. Protein size was evaluated relative to the Protein Plus Dual Color Ladder (Biorad, 1610374). Please see Supplementary Fig. 11 for the full blot shown in Fig. 7A.

#### Immunocytochemistry

Detection of GFP, OCT4, NANOG and TUJ1 was performed using standard methods. In brief, cells were fixed in 4% PFA overnight at 4 °C, and targets detected using the following primary antibodies: GFP at 1:400 (Aves Labs, Aves-1020), OCT4 at 1:200 (BD Biosciences, 611202), NANOG at 1:200 (Abcam, ab21603), and TUJ1 at 1:1000 (Abcam, ab18207). For fluorescent detection, the following secondary antibodies were used at 1:400: Alexa Fluor 488-Donkey Anti-Chicken IgY (Jackson ImmunoResearch, 703-546-155), Alexa Fluor 594 Goat^Mouse (ThermoFisher, A21125), Alexa Fluor 594 Goat^Rabbit (Invitrogen, A11012) and Alexa Fluor 488 Goat^Rabbit (Invitrogen, A32731TR).

Embryos were fixed in 4% PFA overnight at 4 °C, and targets detected using the following primary antibodies: GFP at 1:400 (Aves Labs, Aves-1020), Cyclin E1 at 1:200 (R&D Systems, AF6810), Cyclin B1 at 1:200 (Cell Systems Technology, 4138). For fluorescent detection, the following secondary antibodies were used at 1:400: Alexa Fluor 488-Donkey Anti-Chicken IgY (Jackson ImmunoResearch, 703-546-155), Alexa Fluor 488 Donkey^Rabbit IgG (ThermoFisher, A21206), and Alexa Fluor 594 Donkey^Sheep IgG (ThermoFisher, A11016). The fraction of cyclin E1+ and cyclin B1+ cells at each embryonic stage was quantified by an experimentally blinded individual. Cells were scored cyclin B1+ when either nuclear or cytoplasmic cyclin B1 was detected, and cyclin E1+ when nuclear cyclin E1 exceeded background.

#### Flow cytometry

To measure pRb over the course of the cell cycle, we simultaneously stained for pRb 807/811 and DNA content. In brief, samples were dissociated to single-cell suspensions, split into samples of 1 million cells and fixed in 4% PFA for 10 min with light agitation at room temperature. Cells were permeabilized for 10 min with methanol at −20 °C. Cells were then rinsed in 10% normal goat serum (NGS) and 2% bovine serum albumin (BSA) in PBS. We stained for pRb 807/811 using a 1:50 dilution of PE-conjugated antibody (Cell Signaling Technologies, D20B12) in +5% NGS, +5% BSA, and 0.2% Triton-X (Tx). Cells were then rinsed, resuspend in 0.5 ml of FxCycle Violet (ThermoFisher, F10347), filtered through a 40 μm filter, and analyzed after 15–30 min of incubation.

To measure Cyclin E1 and Cyclin B1 across the cell cycle, we co-stained for both cyclins as well as DNA content. In brief, samples were dissociated to single-cell suspensions, and 0.5 million cells fixed in 4% PFA for 30 min on ice with light agitation. Cells were then blocked and permeabilized in 5% NGS, 5% BSA and 0.2% Triton-X in PBS (blocking buffer). We stained for Cyclin E1 using a 1:100 dilution of antibody (Cell Signaling Technologies, 20808S) in blocking buffer. Alexa fluor 647 (ThermoFisher, A21245) was used at 1:200 to detect Cyclin E1, and Alexa fluor 488 (Jackson ImmunoResearch, 115-545-205) was used at 1:200 to detect Cyclin B1. Cells were then rinsed, resuspend in 0.5 ml of FxCycle Violet (ThermoFisher, F10347), filtered through a 40um filter, and analyzed after 15–30 min of incubation.

### Additional statistical analysis

ANOVAs and adjusted *t* tests of cellular data were performed using PRISM v9. *T* tests of expression, Kolmogorov–Smirnov tests, Wilcoxon Rank-Sum tests, Pearson and Spearman’s correlations via the asymptotic *t* approximation were performed with R v3.5.1.

### Reporting summary

Further information on research design is available in the Nature Research Reporting Summary linked to this article.

## Supplementary information

Supplementary Information

Description of Additional Supplementary Files

Supplementary Data 1

Supplementary Data 2

Supplementary Data 3

Supplementary Data 4

Supplementary Data 5

Supplementary Data 6

Supplementary Data 7

Supplementary Data 8

Reporting Summary

## Data Availability

All raw sequencing data and processed counts data generated in this study have been deposited in the GEO database under the accession code GSE142215 (https://www.ncbi.nlm.nih.gov/geo/query/acc.cgi?acc=GSE142215). There are no restrictions on availability of the data. To analyze these data, we used Mus musculus genome assembly GRCm38 mm10 (https://www.ncbi.nlm.nih.gov/assembly/GCF_000001635.20/), the comprehensive GRCm38 GENCODE gene annotation (https://www.gencodegenes.org/mouse/release_M2.html), and Cyclebase (https://cyclebase.org/CyclebaseSearch). Source data are provided with this paper.

## Source data

Source Data
