# Supplementary material for: G1/S restriction point coordinates phasic gene expression and cell differentiation
Source: Nat Commun. 2022 Jun 27;13:3696. doi: 10.1038/s41467-022-31101-0 (PMC9237072; doi:10.1038/s41467-022-31101-0)
Supplement: Supplementary file 1 — Supplementary Information [file 41467_2022_31101_MOESM1_ESM.pdf]

Supplementary Information for

G1/S restriction point coordinates phasic gene expression and cell differentiation

DeVeale et. al. 2022

Supplementary Figures 1-11

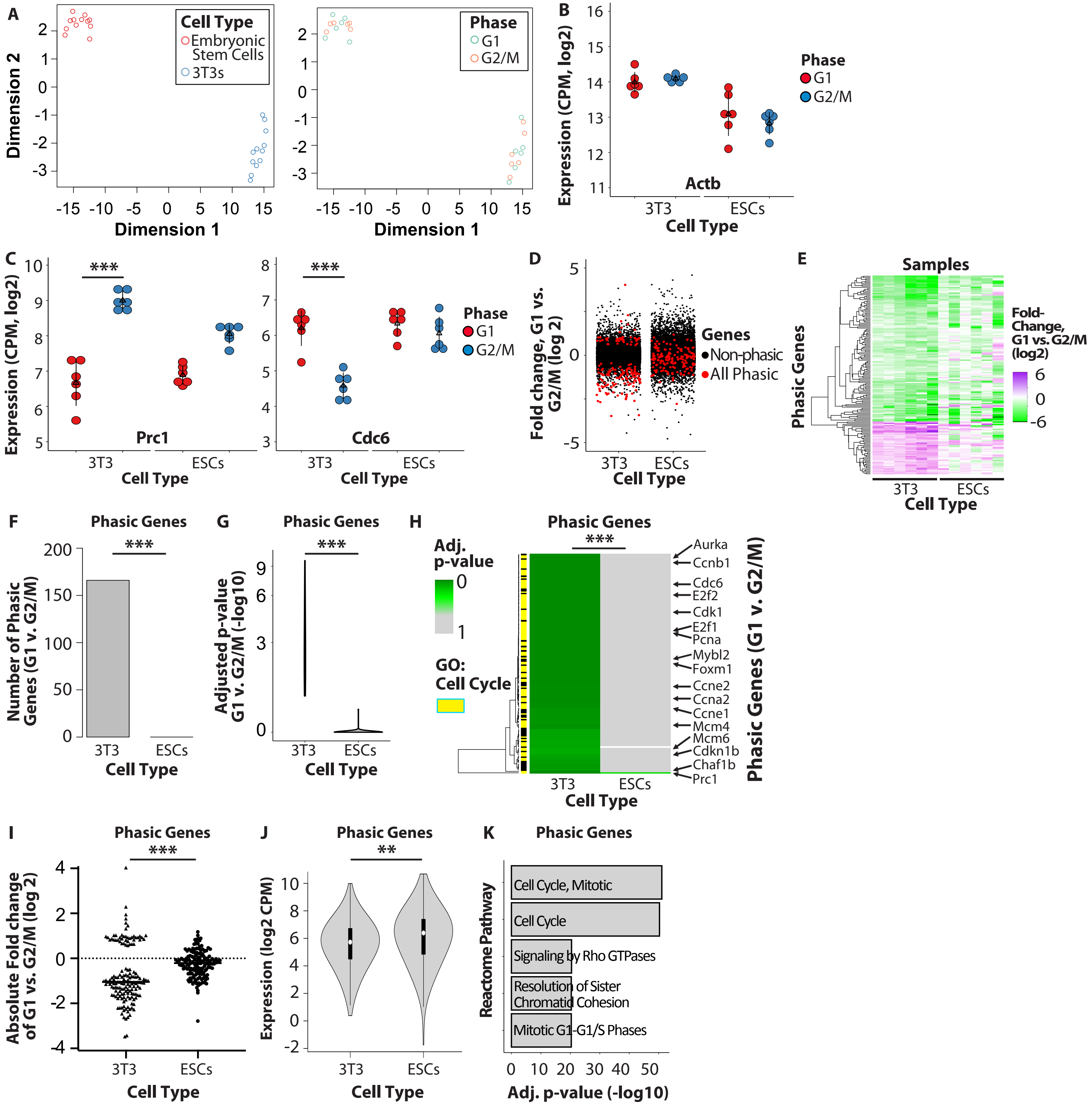

Supplementary Figure 1.

Supplementary Figure 1. Phasic expression in somatic and pluripotent cells. (A) Uniform Manifold Approximation and Projection (UMAP) plots of each replicate separated by cell type (left panel) and cell cycle phase (right panel). (B) The expression of *Actb* in each cell type. Black dot, mean. Black line, SD. n=6 biologically independent samples for each cell type and phase. (C) The expression of *Prc1* (left panel, *** Benjamini-Hochberg (BH) adjusted *P*-value (adj. *P*)=1.09e-13) and *Cdc6* (right panel, *** BH adj. *P*=4.88e-8) in each cell type. Black dot, mean. Black line, SD. n=6 biologically independent samples for each cell type and phase. (D-K) Comparison of 'phasically expressed' genes, those differentially expressed between G1 and G2/M of either 3T3 or mouse embryonic stem cells, across cell types. (D) The log2 fold-change between G1 and G2/M of all expressed genes (black dots) and phasically expressed genes (red dots, adj. *P* <0.1) for each cell type. (E) The log2 fold-change between G1 and G2/M of phasically expressed genes in each replicate. (F) More genes are differentially expressed between G1 and G2/M (adj. *P* <0.1) of somatic 3T3s than pluripotent ESCs (****P* <0.001, Chi-square test). (G) The distribution of adj. *P*-values (from 'F') in each cell type (Wilcoxon Rank Test, two-sided, ****P*<0.001). (H) Heat map of adj. *P*-values for all genes shown in G (Wilcoxon Rank Test, two-sided, ****P*<0.001). (I) The distribution of fold change values for all genes shown in G (****P* <0.001, two-tailed t-test). (J) The distribution of log2 counts per million for all genes from F (Wilcoxon Rank Test, two-sided, ***P*<0.0081). White dot, median. Box edges, 25th and 75th quartiles. Whiskers, 1.5x the IQR of the box edge. (K) The most enriched Reactome pathways among the union of genes phasically expressed between E7.5 and E9.5 evaluated using EnrichR [1]. (B-J) Differential expression was evaluated with a Wald Chi-squared test and adjusted for multiple tests using the Benjamini-Hochberg approach, n=6 biologically independent samples for each cell type and phase (each dot represents an individual sample in B,C). The source data for E and G are provided in "Supplementary Source Data.xlsx".

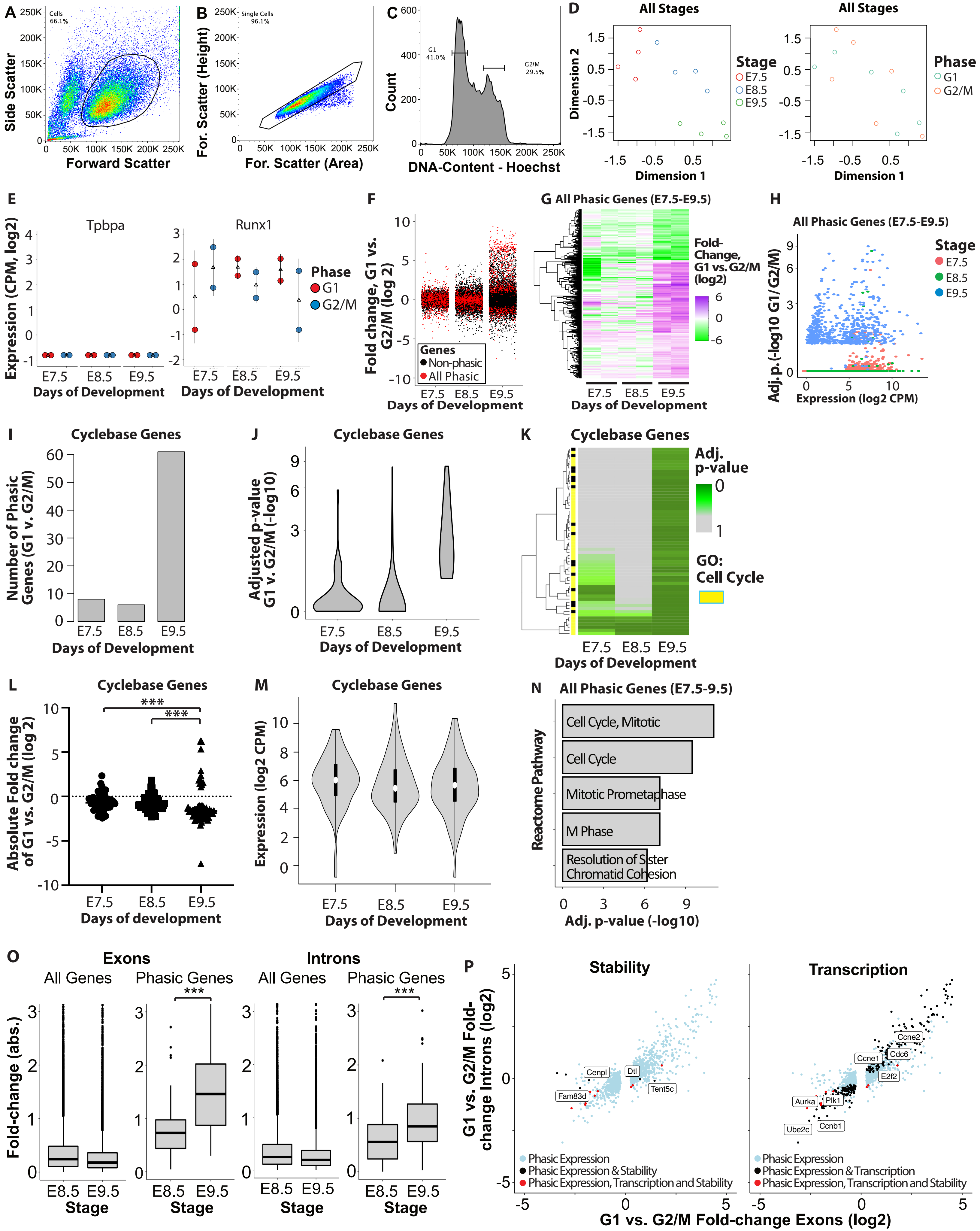

Supplementary Figure 2.

Supplementary Figure 2. Gain of phasic expression following gastrulation. (A-C) Representative FACS strategy. (A) Forward/side scatter gating cells from debris. (B) Forward area/height scatter gating single-cells from doublets. (C) Gating of G1 and G2/M phase cells based on DNA-content. (D) UMAP plots of replicates labeling developmental stage (left) and cell cycle phase (right). (E) Placental (*Tbpba*) and yolk sac (*Runx1*) marker expression by phase and stage. (F,G) The fold-change of genes phasically expressed at E7.5, E8.5 or E9.5 (1F) for each developmental stage. (H) Scatterplot of BH-adjusted *P*-values for phasic genes (1F) in relation to transcript abundance. (I-N) Phasic expression of Cyclebase genes. (I) The number of Cyclebase genes showing differential expression between G1 and G2/M (BH-adj. P <0.1, Wald Chi-squared test) at each developmental stage. Among expressed Cyclebase orthologs, 3.2% (n=8/251) are phasic at E7.5, 2.8% (n=7/251) at E8.5 and 24.3% (n=61/251) at E9.5. (J,K) The distribution (J) and heatmap (K) of BH-adjusted *P*-values at each developmental stage for the union of genes in I. (L) The distribution of fold-change values for all genes shown in J (***P <0.001, Tukey's post-hoc, one-way ANOVA). (M) The counts per million distribution for genes in J (n.s., P>0.5, ANOVA). White dot, median. Box edges, 25th and 75th quartiles. Whiskers, 1.5x the IQR of the box edge. (D-M) n=2 biologically independent samples for each stage and phase. (H-K) Differential expression was evaluated with a Wald Chi-squared test and adjusted for multiple tests using the BH approach. (N) The most enriched Reactome pathways among the union of genes phasically expressed between E7.5 and E9.5 [1]. (O) Comparison of distribution of fold-changes of all phasic genes at E8.5 and E9.5 as measured by either exon reads (***P=3.10e-14, two-tailed t-test) or intron reads (***P=1.68e-05, two-tailed t-test). Centre, median. Bounds of box, 25th and 75th percentiles. Whiskers, the minima and maxima are the most extreme points within 1.5x the IQR of the box edge. n=1 (E8.5) and n=3 (E9.5) biologically independent litters. Litters of 6 embryos at E8.5, and 4, 7 and 3 embryos at E9.5 were pooled. (P) Cell cycle regulated transcription rather than stability is responsible for phasic expression at E9.5. (Left) Among phasically expressed genes at E9.5 (BH-adj. P <0.05), only 9 are differentially stable and not differentially transcribed between G1 and G2/M (BH-adj. P <0.05, subset are labeled). (Right) 277 phasically expressed genes are differentially transcribed and not differentially stable between G1 and G2/M (BH-adj. P <0.05, subset are labeled). The fold-change of exons and introns among all phasically expressed genes are correlated (r=0.84, P=2.2e-16, Pearson). 60.34% (n=143/237) of expressed Cyclebase genes are phasically expressed at E9.5. "Supplementary Source Data.xlsx" provides the source data for A-C, G-I, J and L.

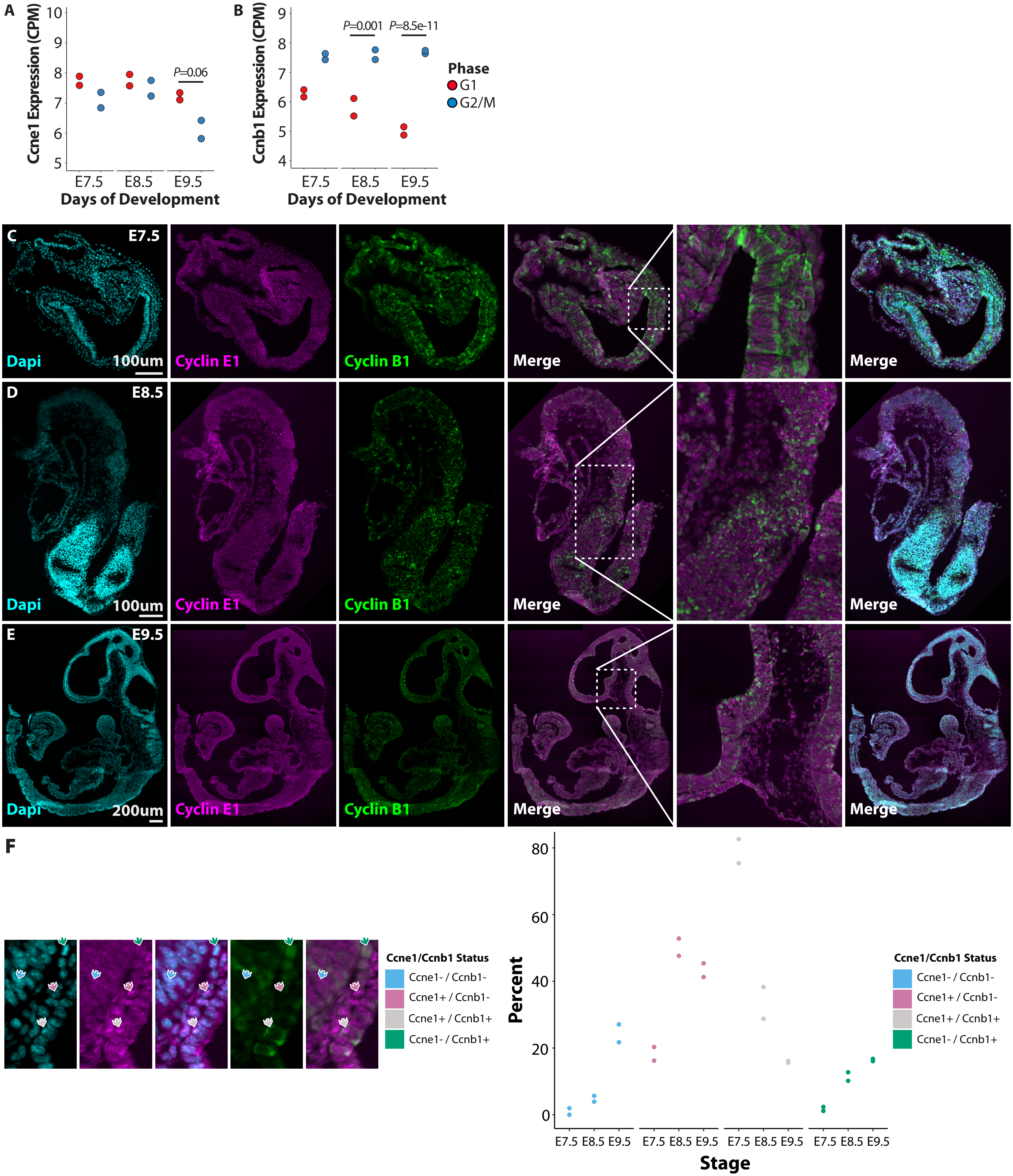

Supplementary Figure 3.

Supplementary Figure 3. The relative distribution of Cyclin E1 and Cyclin B1 at E7.5, E8.5 and E9.5. (A,B) Phasic expression of *Ccne1* and *Ccnb1* increases between E7.5 and E9.5. The transcript expression of *Ccne1* (A) and *Ccnb1* (B) in G1 and G2/M between E7.5 and E9.5 of development (n=2 biologically independent litters. Litters of 6 and 4 embryos at E7.5, 8 and 6 embryos at E8.5, and 3 and 5 embryos at E9.5 were pooled. Differential expression was evaluated with a Wald Chi-squared test and n=2 for each stage and phase (each dot represents an individual sample in A,B). The source data is provided in "Supplementary Source Data.xlsx". (C-E) Co-staining of Cyclin E1 and Cyclin B1 in E7.5 (C), E8.5 (D), and E9.5 (E) embryos. (F) The relative distribution of Cyclin E1 and Cyclin B1 protein in E7.5, E8.5 and E9.5 embryos. (Left panel) An inset from the E9.5 embryo showing representative examples of each quantified category. (Right panel) The fraction of cells expressing both Cyclin E1 and Cyclin B1 declines between E7.5 and E9.5 (**P=0.002, one-way ANOVA; n=521 (E7.5), n=680 (E8.5), n=1516 (E9.5) biologically independent cells).

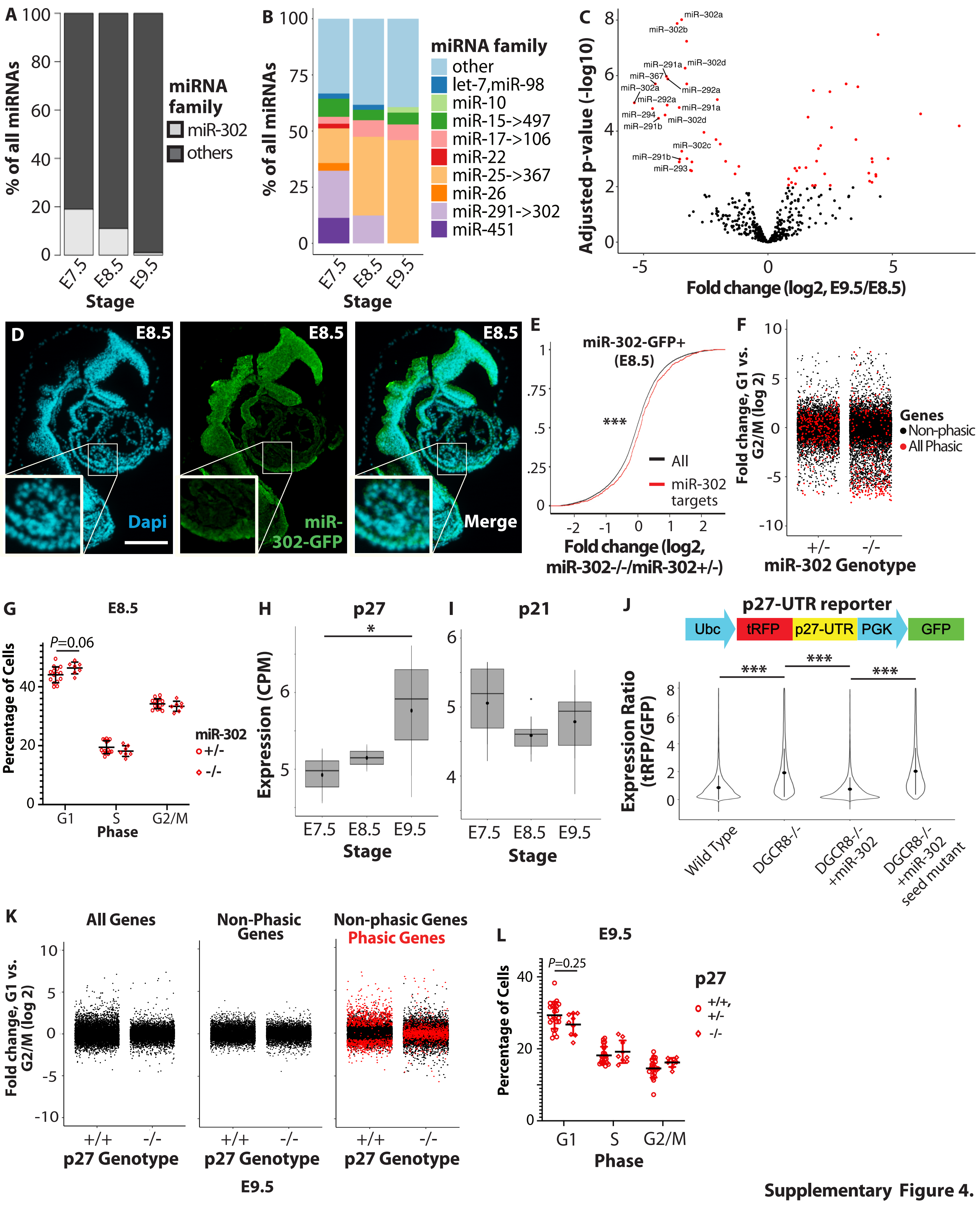

Supplementary Figure 4.

Supplementary Figure 4. Analysis of *mir-302*/P27 axis. (A) Percent miR-302a-d among entire miRNA pool at each developmental stage as determined by small RNA-sequencing. (B) Percent individual miRNA families contribute to the entire miRNA pool. Only families ≥2% of the total miRNA pool are plotted. (C) Differential expression of miRNAs between E9.5 and E8.5 embryos. Differentially expressed miRNAs are depicted in red (adj. *P* <0.01, BH-adjusted empirical Bayes moderated t-statistic). (D) GFP immunofluorescence in E8.5 *mir-302*-GFP reporter mice shows its heterogeneous expression (scale bar = 200um). (E) Cumulative fold-change distribution in gene expression when comparing *mir-302*-GFP sorted cells from control and *mir-302*-/- E8.5 embryos. Black line, all genes, Red line, Targetscan predicted *mir-302* targets (Wilcoxon Rank-Sum test, two-sided, ****P*=0.0001). Note shift of *mir-302* targets to right shows their upregulation in knockouts relative to controls. (F) The log2 fold-change between G1 and G2/M of all expressed genes (black dots) and phasically expressed genes (red dots, adj. *P* <0.1) in miR-302-/- and control samples. 19 Cyclebase genes were phasically expressed in controls (7.8%), while 28 genes were phasically expressed in miR-302-/- (11.5%). (G) The distribution of cells across the phases of the cell cycle in control and *mir-302*-/- E8.5 embryos (G1 *P*=0.062, S *P*=0.46, G2/M *P*=0.77, Sidak's multiple comparison test, two-way ANOVA). Center line, mean. Upper/Lower lines, SD. n=15 (*mir-302*+/-), n=7 (*mir-302*-/-) biologically independent embryos. (H) The abundance of p27 transcripts at each developmental stage (E7.5 vs. E8.5 *P*=0.78, E7.5 vs. E9.5 *P*=0.05, Dunnett's multiple comparison, one-way ANOVA). (I) The abundance of p21 transcripts at each developmental stage (E7.5 vs. E8.5 *P*=0.27, E7.5 vs. E9.5 *P*=0.17, Dunnett's multiple comparison, one-way ANOVA). (H,I) Centre, median. Bounds of box, 25th and 75th percentiles. Whiskers, the minima and maxima are the most extreme points within 1.5x the IQR of the box edge. (J) Reporter analysis of miR-302 targeting of the p27 3'UTR. (Top) Reporter construct schematized. (Bottom) Violin plot of relative tRFP/GFP in individual cells carrying reporter in WT vs. *Dgcr8-/-* background or *Dgcr8-/-* ± miR-302 or seed mutant mimic (****P* <0.001, Tukey's post-hoc, one-way ANOVA). Dot, mean. Line, SD. (K) The log2 fold-change in expression between G1 and G2/M of p27 mutant and control E9.5 embryos. (Left panel) All expressed genes. (Middle panel) Only non-phasic genes. (Right panel) Expressed non-phasic genes (black dots) and phasically expressed genes (red dots, adj. *P* <0.1). 29.2% (66) Cyclebase genes were phasically expressed in controls, while 11.5% (26) were phasically expressed in p27-/-. (L) The distribution of cells across the phases of the cell cycle in control and p27-/- E9.5 embryos (G1 *P*=0.25, S *P*=0.94, G2/M *P*=0.95, Sidak's multiple comparison, two-way ANOVA). Center line, mean. Upper/Lower lines, SD. p27+/+,+/- (n=23 biologically independent embryos), p27-/- (n=9 biologically independent embryos).

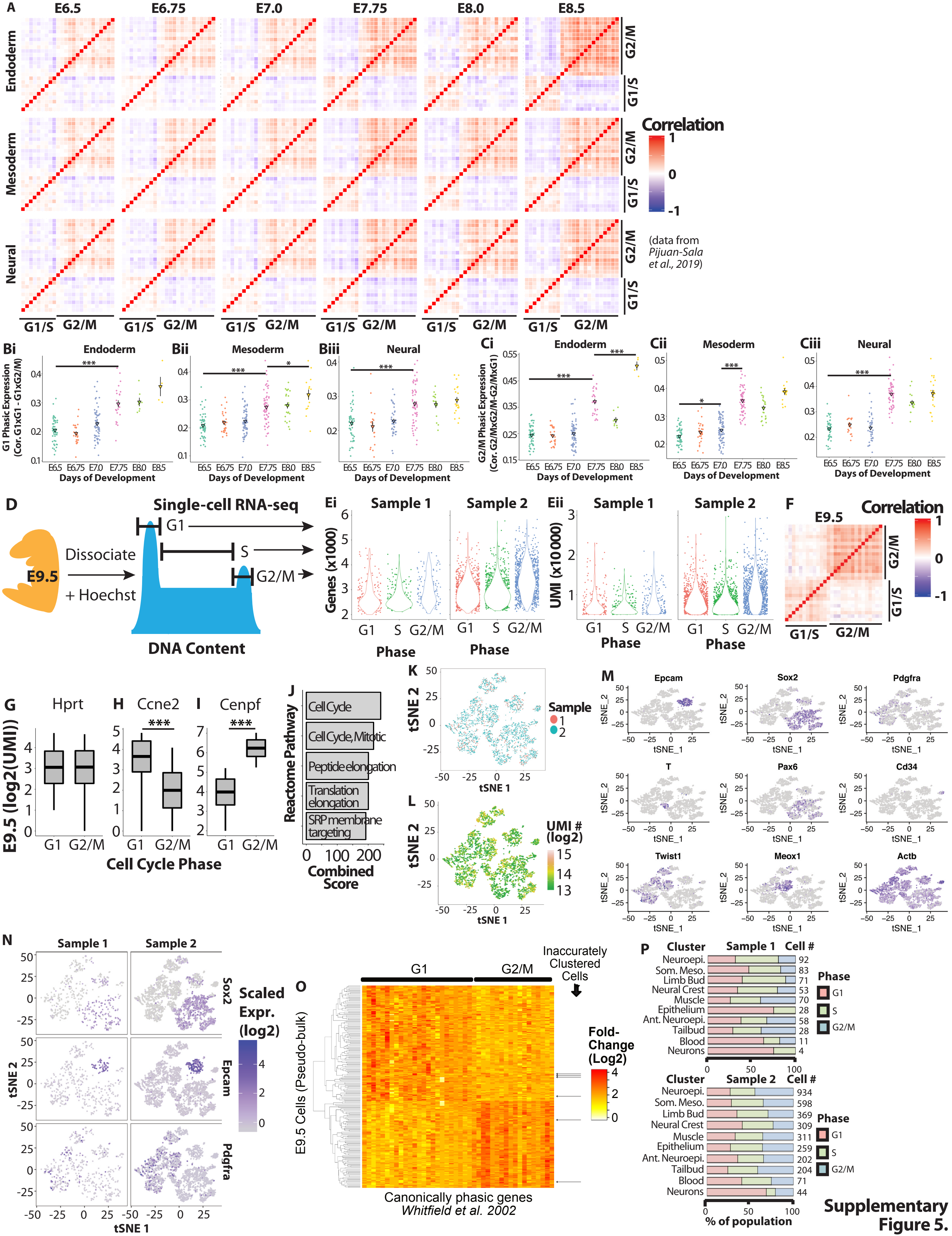

Supplementary Figure 5.

Supplementary Figure 5. Lineage and phasic expression analysis at E9.5. (A-C) The intra-phase correlation of canonical cell cycle regulated genes indicates increased phasic expression between E6.5 and E8.5 of embryogenesis. (A) Gene-gene Pearson correlation using a developmental atlas of single-cell profiles [2] and canonically phasic genes [3]. The annotated peak expression phase is labeled. Note increased co-expression of genes within G1/S and G2/M cells at E8.5, relative to E6.5. (B,C) The relative intra-phase correlation of canonical cell cycle regulated genes. G1 phasic expression evaluated as the correlation among G1-S genes relative to the correlation between G1-S and G2/M genes (see methods). The correlation among G1-S genes increases relative to G2/M genes during development of different lineages (**P*<0.05, ***P*<0.01, ****P*<0.001, Tukey's multiple comparison, two-way ANOVA, the number of biologically independent cells at each stage (E6.5, E6.75, E7.0, E7.75, E8.0, E8.5), n=2667, 1197, 8774, 623, 460, 646 (endoderm), n=2671, 1353, 10609, 3246, 5445, 3490 (mesoderm), n=2278, 861,5118, 3290, 4090, 4392 (neural). (C) G2/M phasic expression evaluated as in B. (D) Schematic of single cell workflow. In brief, E9.5 embryos were dissociated and sorted by cell cycle phase based on DNA-content before single cell RNA-seq. (E) Violin plots of single-cell sequencing quality control metrics (genes (i) and unique molecular identifiers (UMI) per cell (ii)). (F) Gene-gene correlation of canonical cell cycle regulated genes at E9.5 [3]. The annotated peak expression phase is labeled. (G-I) *Hprt*, *Ccne2* and *Cenpf* expression in the G1 and G2/M phases of pseudo-bulk derived from single cells at E9.5. BH-adjusted ***FDR=1.22e-38 (*Ccne2*), ***FDR=1.47e-55 (*Cenpf*), Hurdle model (see methods), n=3301 biologically independent cells. Centre, median. Bounds of box, 25th and 75th percentiles. Whiskers, the minima and maxima are the most extreme points within 1.5x the IQR of the box edge. (J) Reactome pathways enriched among genes phasically expressed at E9.5 using EnrichR [1]. (K) A tSNE representation of the two independent single cell samples colored by sample. (L) Same as 'K,' but cells are color coded for number of UMIs/cell. (M) Expression of lineage markers among the eleven tSNE clusters. (N) Distribution of lineage markers among the t-SNE clusters, shown for each sample separately. (O) Unsupervised clustering of differential expression of the canonically phasic genes between G1 and G2/M of pseudobulk cells by Euclidean distance [3]. Genes (x-axis) are ordered by phase while the cell clustering (y-axis) is unsupervised. Arrows indicate cells where DNA content and unsupervised clustering disagreed. Note that vast majority of cells clustered correctly to phase (as defined by DNA content) confirming establishment of classic phasic expression at this stage and power of the pseudobulk approach. (P) Distribution of cells across cell cycle phases in each lineage cluster as well as the total number of cells in each.

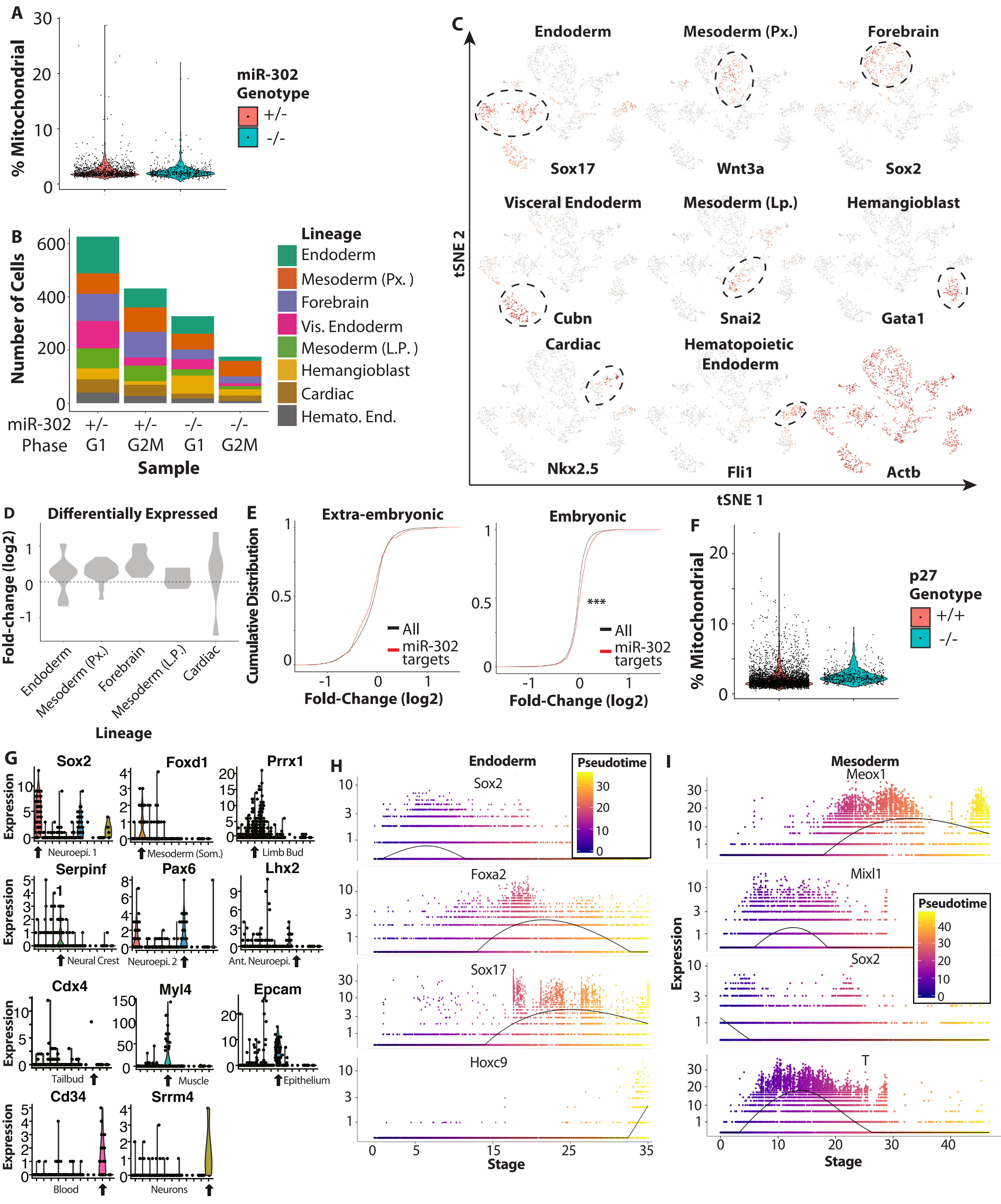

Supplementary Figure 6.

Supplementary Figure 6. Analysis of single cells in E7.5 *mir-302* and E9.5 p27 knockout embryos. (A) The percentage of mitochondrial reads per cell in mir-302 mutant and control cells. 3.42% (n=38/1111) of cells exceed the 5% mitochondrial read threshold used for filtering in *mir-302*+/- compared to 3.28% (n=17/518) in *mir-302*-/-. (B) Number of E7.5 single-cell transcriptomes-profiles captured with ≥5000 UMIs, organized by genotype and cell cycle phase. (C) Expression of lineage markers among t-SNE clusters of E7.5 *mir-302*+/- and *mir-302*-/- embryos. (D) Fold-change of *mir-302* targets that are differentially expressed by lineage (*P* <0.1). (E) Cumulative distribution plot of log2 fold-change in gene expression when comparing the cells control and *mir-302*-/- E8.5 embryos. The lineage depicted is labeled in each panel. Black line, all genes, Red line, Targetscan predicted *mir-302* targets. Note the significant shift of *mir-302* targets to the right in embryonic but not extra-embryonic cells, consistent with ongoing targeting in embryonic cells (Wilcoxon Rank-Sum test, two-sided, ****P* <0.001). (F) The percentage of mitochondrial reads per cell in p27-/- and control cells. 0.57% of control E9.5 cells exceed the 5% mitochondrial read threshold used for filtering (n=19/3309), compared to 4.34% of p27 cells (n=23/530). (G) Lineage marker expression in p27-/- single-cell transcriptomes across populations inferred from control cell clusters confirming the alignment of cells from p27-/- with the reference wt E9.5 populations using the mutual nearest neighbor approach. (H,**I**) Simultaneous analysis of pseudotime and phasic expression in individual cells. (H) Expression of marker genes in pseudotime of endoderm lineage. The decline in pluripotency marker *Sox2* is followed by the early rise of canonical mesoderm markers *Foxa2* and *Sox17* before later expression of *Hoxc9*. (I) Expression of marker genes in pseudotime of mesoderm lineage. The decline in pluripotency marker *Sox2* is followed by the early rise of canonical mesoderm markers *T* and *Mixl1* before later expression of *Meox1*.

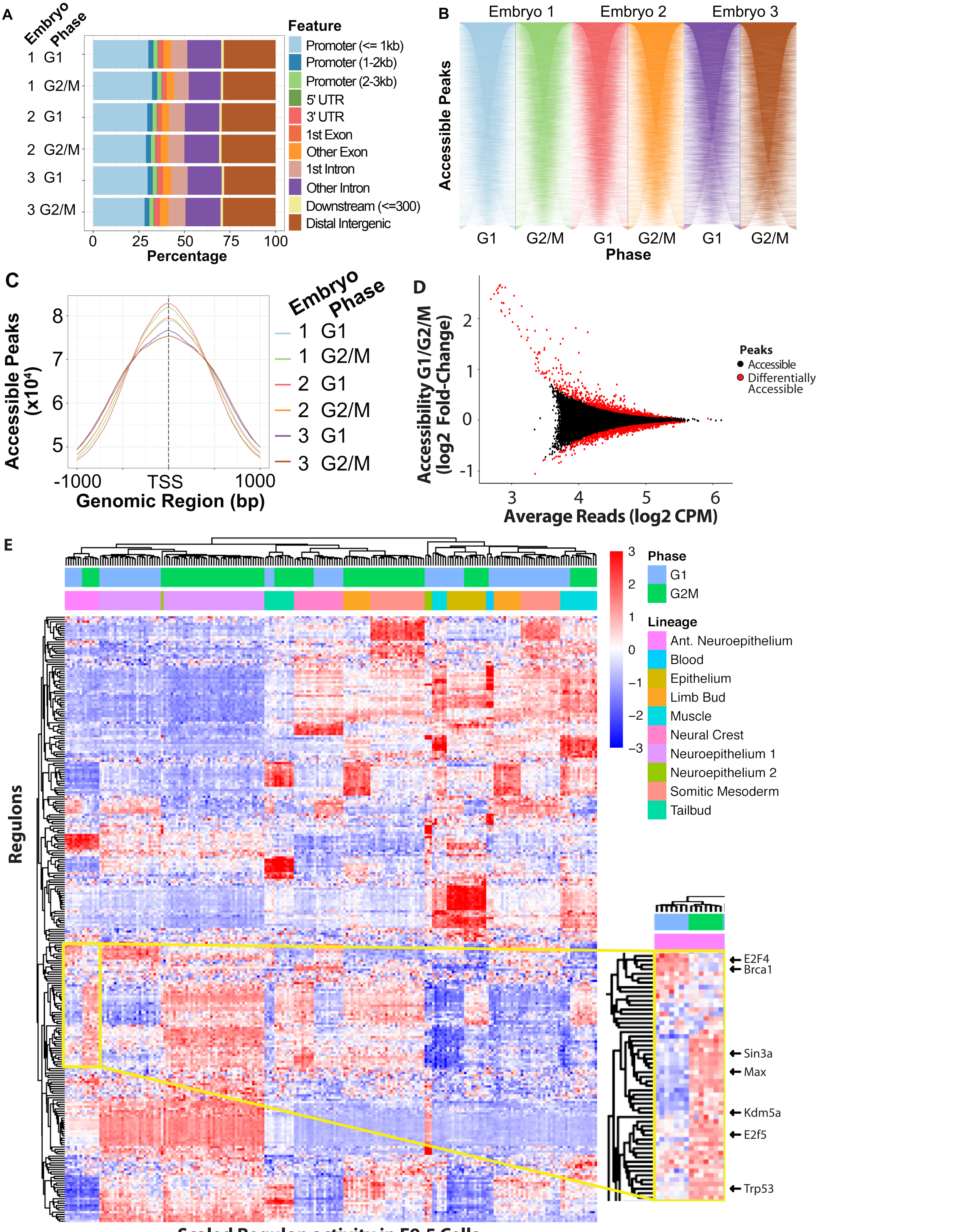

Supplementary Figure 7.

Supplementary Figure 7. Quality metrics of ATAC-seq data on cell cycle phase-sorted cells from E9.5 embryos. (A) The distribution of accessible chromatin peaks (FDR<0.1) across genomic features. (B) Heatmap of ATAC-seq reads relative to TSS. (C) The collective frequency of reads at accessible peaks centered on TSS. (D) Differential accessibility between G1 and G2/M of E9.5 embryos (ATAC-seq) in relation to the magnitude of accessibility. Peaks that show differential expression (adj. P <0.01, Benjamini-Hochberg adjusted empirical Bayes moderated t-statistic) are shown as red dots. The source data is provided in "Supplementary Source Data.xlsx". (E) All regulons identified by SCENIC among phasically expressed genes at E9.5. Unsupervised clustering of the regulons among E9.5 pseudobulk cells.

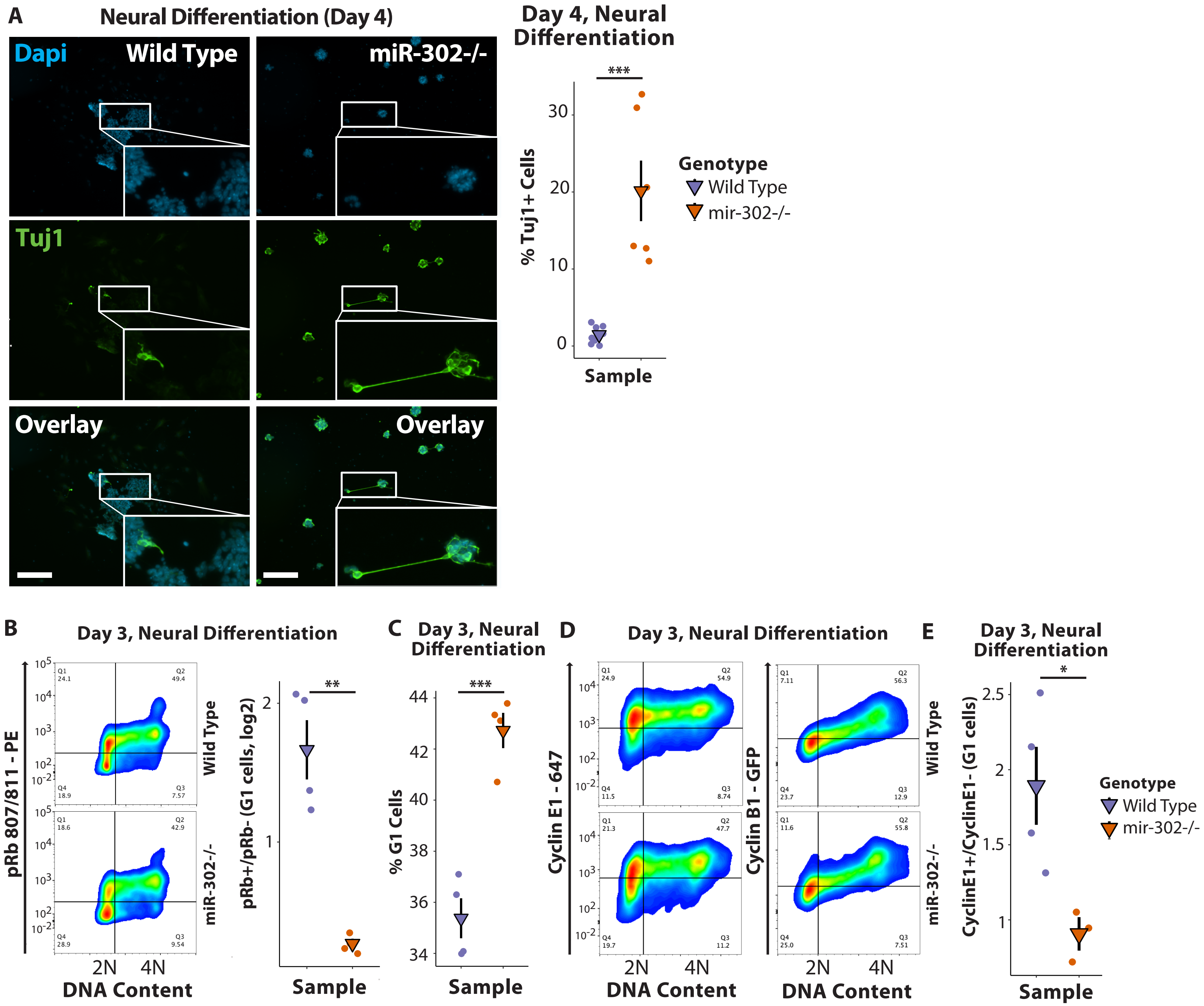

F

Cell line	Δ size (bp)	Δ encompass entire ESCC target site? (Y/N)	Deleted seed positions
p21-3'UTR+/Δ302 #1	51	Y	all
p21-3'UTR+/Δ302 #2	13	Y	all
p21-3'UTR+/Δ302 #3	37	Y	all
p21-3'UTR+/Δ302 #4	5	N	1-5
p21-3'UTRΔ/Δ302 #1	28	Y	all
p21-3'UTRΔ/Δ302 #2	20, 4	Y, N	all, 2-5
p21-3'UTRΔ/Δ302 #3	20, 50	Y	all
p21-3'UTRΔ/Δ302 #4	36	Y	all
p21-3'UTRΔ/Δ302 #5	39	Y	all
p21-3'UTRΔ/Δ302 #6	39	Y	all
p27-3'UTR+/Δ302 #1	49	Y	all
p27-3'UTR+/Δ302 #2	17	Y	all

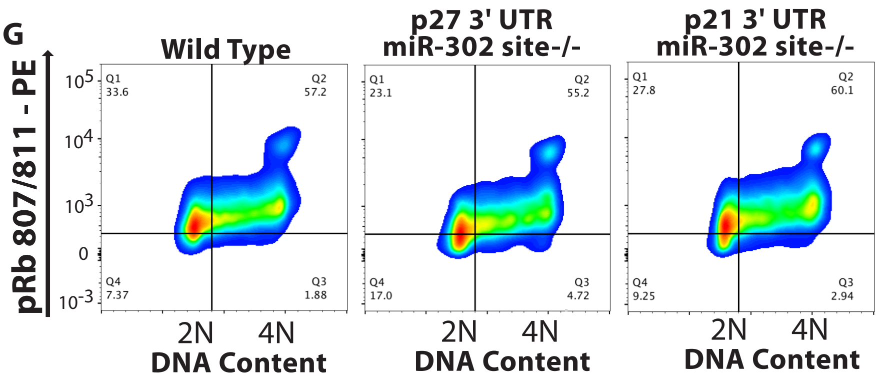

Supplementary Figure 8.

Supplementary Figure 8. *mir-302* target site deletions in the 3'UTR of p27 and p21. (A) Following 4 days of neural differentiation, *mir-302*-/- cells yield a higher frequency of Tuj1+ neurons than control cells. (left panel) βIII-tubulin staining (scale bar = 200um). (right panel) The percentage of βIII-tubulin+ cells per field of view (two tailed t test ****P*=0.00014, n=6 independent fields of view from 2 independent experiments). (B) Rb phosphorylation is reduced during neural differentiation of *mir-302*-/- cells. The fraction of pRb 807/811+ cells is lower in *mir-302*-/- cells than controls after 3 days of differentiation (two-tailed t test ***P*=0.0048, n=4 biologically independent samples). (C) 3 days into neural differentiation, the fraction of *mir-302*-/- cells in G1 phase of the cell cycle is increased relative to controls (two-tailed t test ****P*=0.00041, n=4 biologically independent samples). (D,E) Cyclin E1 prematurely varies during neural differentiation of *mir-302*-/- cells. (D) Flow plots of Cyclin E1 (left column) and Cyclin B1 (right column) in relation to DNA content on day 3 of neural induction. (E) The fraction of G1 Cyclin E1+ cells is lower in *mir-302*-/- cells than controls (two-tailed t test **P*=0.03, n=3 biologically independent samples (day 3)). (F) Details for each independent mutant ESC line harboring deletion of miR-302 target sites within the 3'UTR of *Cdkn1a* (p21) or *Cdkn1b* (p27). (G) Rb phosphorylation is reduced in ESCs harboring deletion of miR-302 target sites within the 3'UTR of *Cdkn1a* (p21) or *Cdkn1b* (p27). The source data for A-C, and E are provided in "Supplementary Source Data.xlsx".

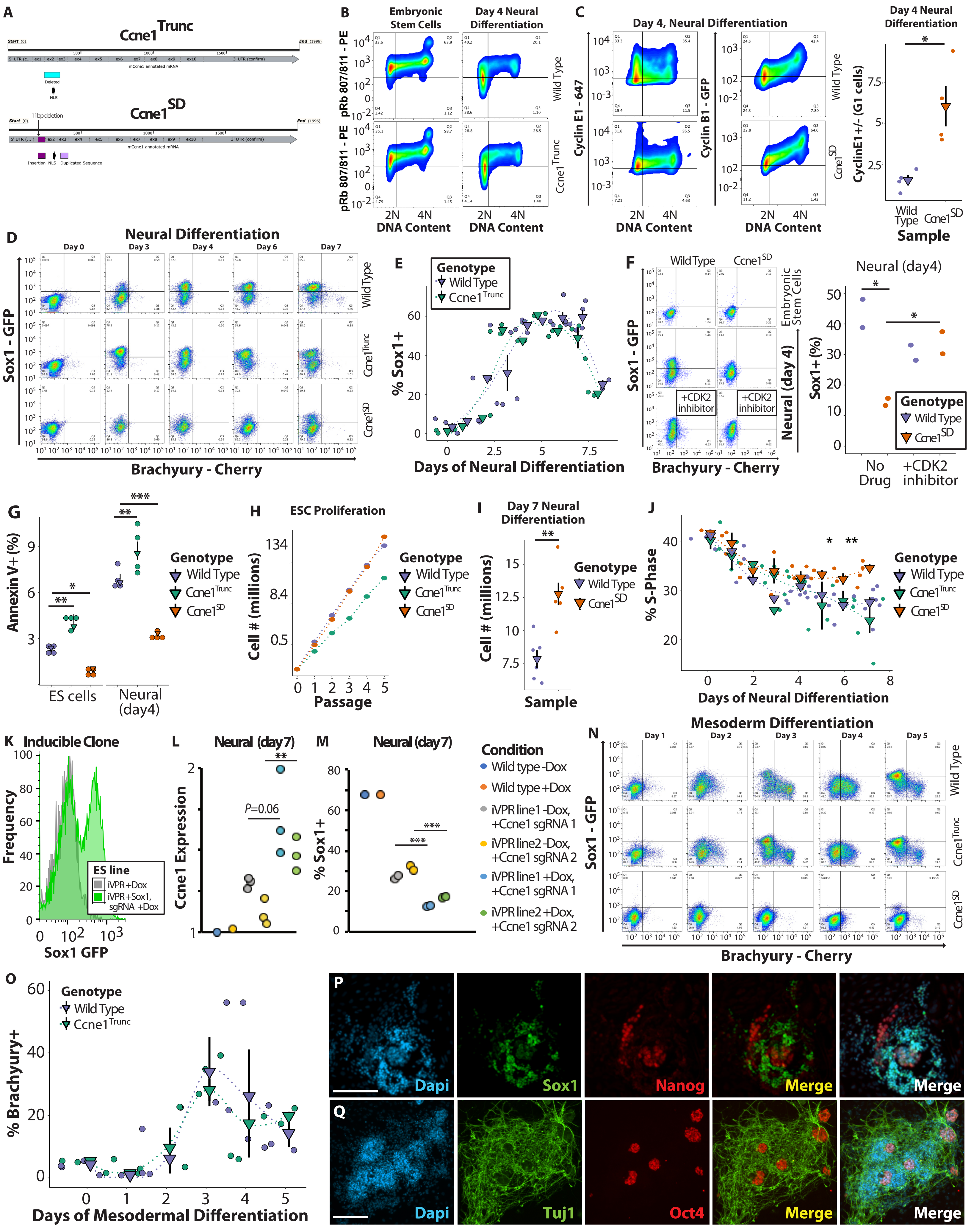

Supplementary Figure 9.

Supplementary Figure 9. Analysis of Ccne1^{SD} and Ccne1^{Trunc} mutant lines. (A) Ccne1^{SD} and Ccne1^{Trunc} mutation schematics. (B) Unphosphorylated Rb (pRb-) at Ser807/811 arises during neural differentiation (day 4) of control and *Ccne1^{Trunc}* mutants. (C) Cyclin E1 persists across the cell cycle of Ccne1^{SD} cells after 4 days of neural induction. (left panel) Flow plots of Cyclin E1 and Cyclin B1 in relation to DNA content. (right panel) The fraction of G1 Cyclin E1+ cells is higher in Ccne1^{SD} than controls (two-tailed t-test **P*=0.01, n=4 biologically independent samples). (D) Flow plots of Sox1-GFP expression during neural differentiation timecourse of Ccne1^{SD}, Ccne1^{Trunc} and control cells. (E) Neural differentiation of Ccne1^{Trunc} mutants compared to controls (biologically independent samples on day 0 to 8 of differentiation: n=3, 2, 2, 3, 2, 2, 3, 3, 2). (F) CDK2 inhibition rescues Ccne1^{SD} differentiation. The fraction of Sox1+ Ccne1^{SD} cells following 4 days of neural differentiation was reduced relative to controls, but increased by administration of a CDK2 inhibitor throughout the differentiation (**P* <0.05, Tukey's post-hoc, two-way ANOVA, n=2 biologically independent samples). (G) Ccne1^{SD} mutants undergo less apoptosis than control ESCs and neuroepithelium (**P*=0.024 [ES], ***P*<0.0052 [ES], ***P*<0.0015 [day 4], ****P*<0.001 [day 4], Tukey's multiple comparison test, two-way ANOVA, n=4 biologically independent samples). (H) Expansion of Ccne1^{SD} ESCs is comparable to controls. (I) The number of Ccne1^{SD} cells is greater than controls following 7 days of neural induction (two-tailed t-test ***P*=0.0029, n=6 biologically independent samples). (J) The fraction of Ccne1^{SD} cells in S-Phase increases during neural differentiation (adjusted t-tests, **P*=0.05, ***P*<0.010, n=3 biologically independent samples). (K-M) Forced Cyclin E1 expression suppresses neural differentiation. (K) A Sox1-GFP clone with doxycycline-inducible dCas9-VPR activity. Doxcycline induces Sox1-GFP expression in clone with Sox1 gRNA and inducible dCas9-VPR. (L) Ccne1 QPCR in two dCas9-VPR +Ccne1 gRNA lines (two-tailed t-test ***P*=0.0097, n=3 biologically independent samples). The two lines are distinct dCas9-VPR clones with distinct Ccne1 gRNAs. (M) Differentiation of ESCs to Sox1+ neuroepithelium is impaired by forcing expression of *Ccne1*. The fraction of Sox1+ cells after 7 days of neural differentiation is reduced following induction of *Ccne1* expression by dCas9-VPR (n=2 biologically independent samples). (N) Flow plots of Bry-Cherry expression during mesodermal differentiation timecourse of Ccne1^{Trunc} and control cells. (O) Mesodermal differentiation of Ccne1^{Trunc} mutants compared to controls (n=3 biologically independent samples). (P,Q) Pluripotency and differentiation markers are mutually exclusive following 10 days of neural differentiation. (P) The pluripotency marker NANOG does not overlap early neural marker Sox1 (GFP from the Sox1-GFP reporter is stained as a Sox1 proxy) (scale bar = 100um). (Q) The pluripotency marker OCT4 does not overlap early neuronal marker TUJ1 (scale bar = 100um). "Supplementary Source Data.xlsx" provides the source data for C, E-I, J, L, M and O.

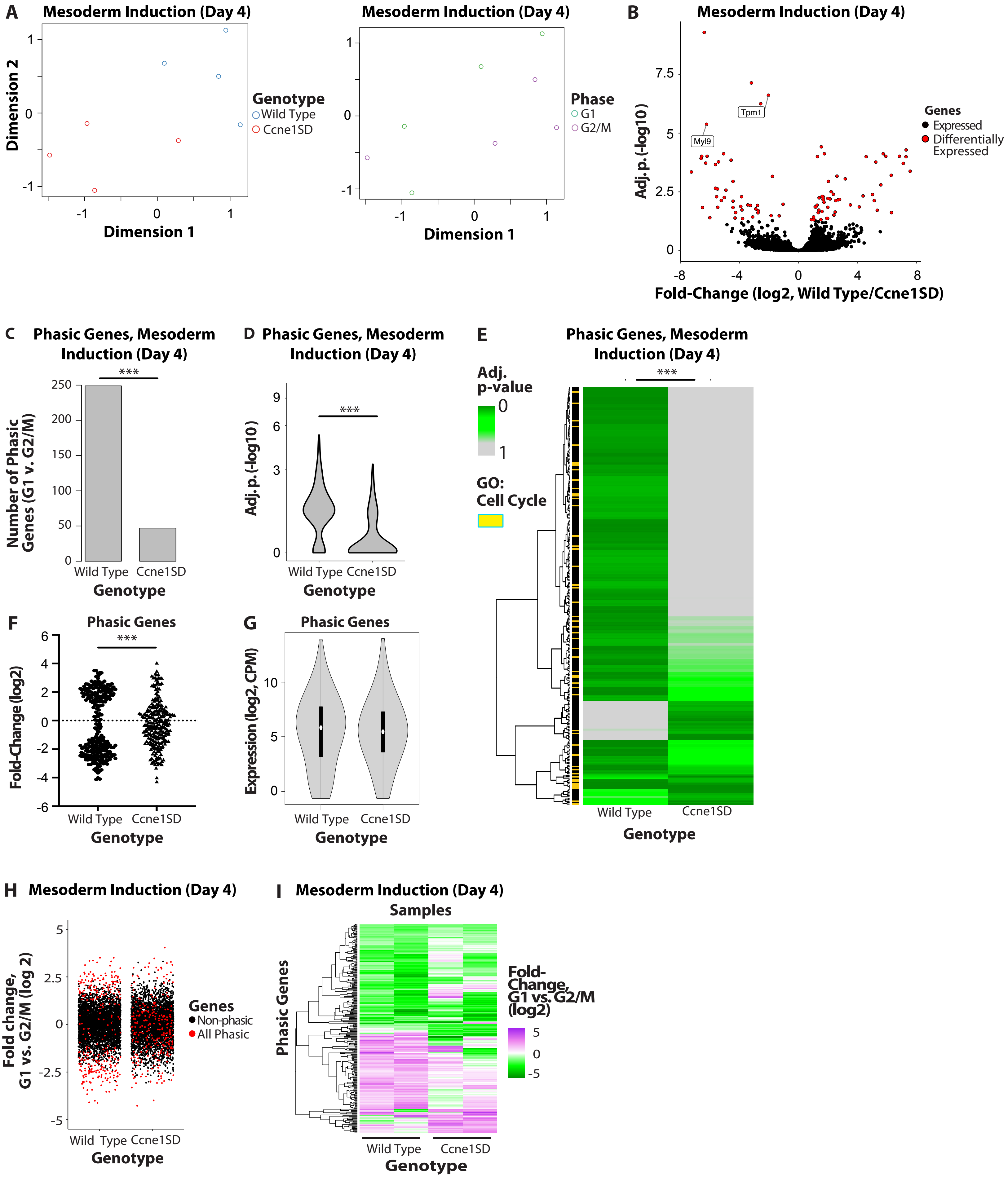

Supplementary Figure 10.

Supplementary Figure 10. Phasic expression in wild type and Ccne1^{SD} cells 4 days into mesoderm differentiation assay. (A) Uniform Manifold Approximation and Projection (UMAP) plots of each replicate separated by genotype (left panel) and cell cycle phase (right panel). (B) Differential expression between wild type and Ccne1^{SD} cells following 4 days of mesodermal induction. The log2 fold-change and -log10 adj. *P*-value of all expressed genes (black dots) and differentially expressed genes (red dots, adj. P <0.05) between genotypes. (C-I) Comparison of 'phasically expressed' genes, those differentially expressed between G1 and G2/M of either wild type or Ccne1^{SD} cells after 4 days of mesodermal induction. (C) More genes are differentially expressed between G1 and G2/M (adj. P <0.1) of wild type than Ccne1^{SD} cells (****P* <0.001, Chi-square test). (D) The distribution of adj. *P*-values (from 'C') in each genotype (Wilcoxon Rank Test, ****P*<0.001). (E) Heat map of adj. *P*-values for all genes shown in C (Wilcoxon Rank Test, ****P*<0.001). (F) The distribution of fold change values for all genes shown in C (****P* <0.001, two-tailed t-test). (G) The distribution of log2 counts per million for all genes from C. White dot, median. Box edges, 25th and 75th quartiles. Whiskers, 1.5x the IQR of the box edge. (H) The log2 fold-change between G1 and G2/M of all expressed genes (black dots) and phasically expressed genes (red dots, adj. P <0.1) for each genotype. (D) The log2 fold-change between G1 and G2/M of phasically expressed genes in each replicate. (C-I) Differential expression was evaluated with a Wald Chi-squared test and n=2 biologically independent samples for each genotype and phase.

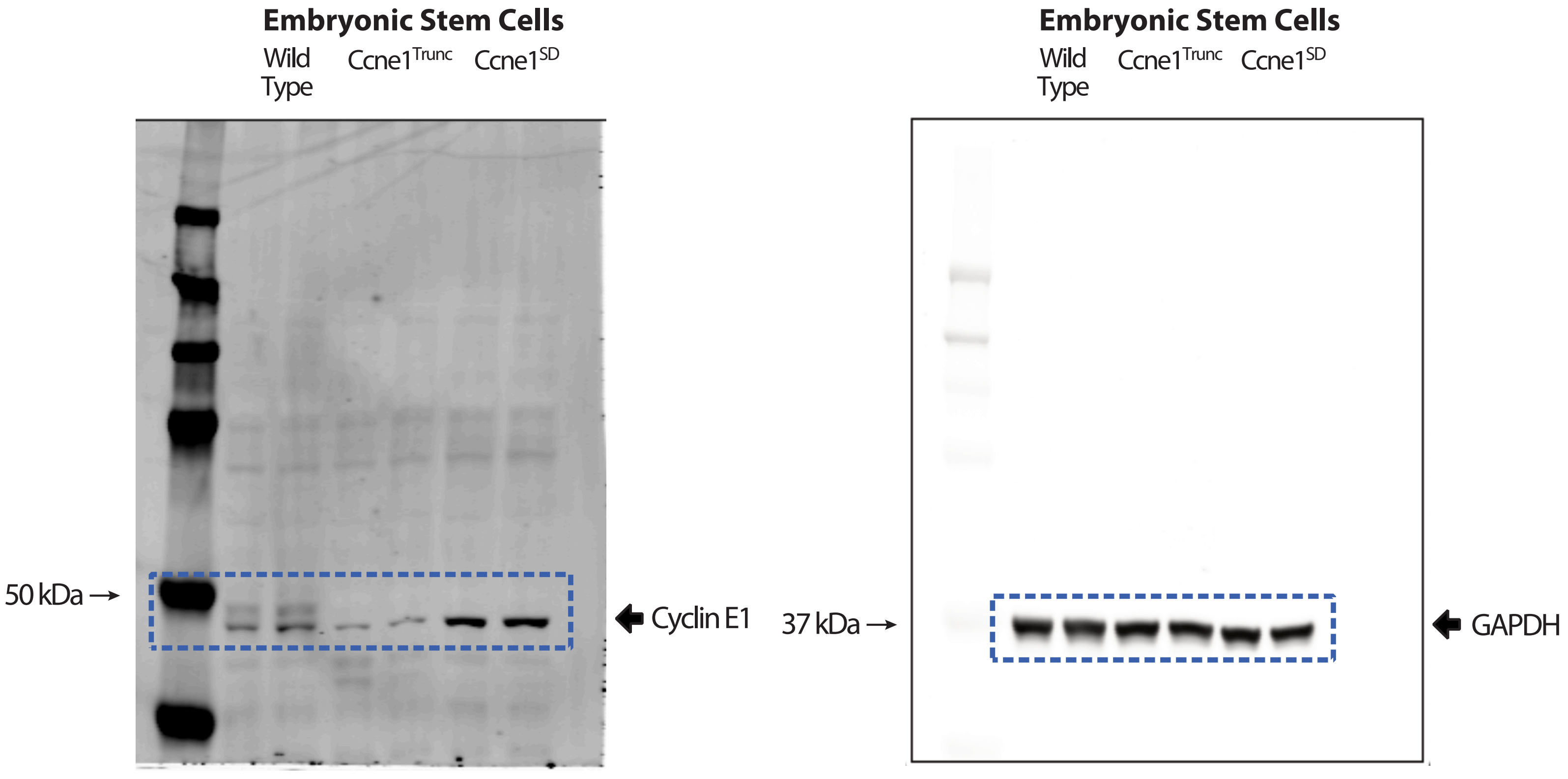

Region cropped and displayed in Figure 7A

Supplementary Figure 11.

Supplementary Figure 11. Whole Western Blots displayed in Figure 7A.

Supplementary References

1. Kuleshov, M.V. *et al.* Enrichr: a comprehensive gene set enrichment analysis web server 2016 update. *Nucleic Acids Res* **44**, W90-7 (2016).
2. Pijuan-Sala, B. *et al.* A single-cell molecular map of mouse gastrulation and early organogenesis. *Nature* **566**, 490-495 (2019).
3. Whitfield, M.L. *et al.* Identification of genes periodically expressed in the human cell cycle and their expression in tumors. *Mol Biol Cell* **13**, 1977-2000 (2002).
