# Supplementary material for: G1/S restriction point coordinates phasic gene expression and cell differentiation
Source: Nat Commun. 2022 Jun 27;13:3696. doi: 10.1038/s41467-022-31101-0 (PMC9237072; doi:10.1038/s41467-022-31101-0)
Supplement: Supplementary file 2 — Description of Additional Supplementary Files [file 41467_2022_31101_MOESM2_ESM.pdf]

Description of Additional Supplementary Files

Supplementary Data 1. **Bulk differential expression between G1 and G2/M of 3T3 and mouse ESCs. 3' end capture.**

Supplementary Data 2. **Bulk differential expression between G1 and G2/M of E7.5, E8.5 and E9.5 embryos. 3' end capture.**

Supplementary Data 3. **Bulk differential expression between G1 and G2/M of E8.5 and E9.5 embryos. Full-length transcript capture.**

Supplementary Data 4. **Bulk differential expression between G1 and G2/M of miR-302-/- (E8.5) and p27-/- (E9.5) and somite-matched control embryos.** GFP+ cells from miR-302-GFP embryos were sorted for the analysis.

Supplementary Data 5. Differential expression between G1 and G2/M of single-cells from E9.5 embryos. Phasic expression across all cells, in individual lineages as well as lineage-specific phasic expression are shown in separate tabs.

Supplementary Data 6. Differential expression between G1 and G2/M in lineages of miR-302-/- (E7.5) and p27-/- (E9.5) embryos along with controls.

Supplementary Data 7. Enrichment of DNA binding motifs in E9.5 G1 and G2/M ATAC-seq peaks evaluated using HOMER.

Supplementary Data 8. Differential expression between G1 and G2/M of control and Ccne1^{SD} cells 4 days into mesoderm differentiation assay. 3' end capture. Phasic expression across control and Ccne1^{SD} cells are shown in separate tabs.
