# Supplementary material for: G1/S restriction point coordinates phasic gene expression and cell differentiation
Source: Nat Commun. 2022 Jun 27;13:3696. doi: 10.1038/s41467-022-31101-0 (PMC9237072; doi:10.1038/s41467-022-31101-0)
Supplement: Supplementary file 11 — Reporting Summary [file 41467_2022_31101_MOESM11_ESM.pdf]

nature research

Corresponding author(s): Robert Blelloch

Last updated by author(s): May 2, 2022

Reporting Summary

Nature Research wishes to improve the reproducibility of the work that we publish. This form provides structure for consistency and transparency in reporting. For further information on Nature Research policies, see our Editorial Policies and the Editorial Policy Checklist.

Statistics

For all statistical analyses, confirm that the following items are present in the figure legend, table legend, main text, or Methods section.

n/a	Confirmed	
☐	☒	The exact sample size (n) for each experimental group/condition, given as a discrete number and unit of measurement
☐	☒	A statement on whether measurements were taken from distinct samples or whether the same sample was measured repeatedly
☐	☒	The statistical test(s) used AND whether they are one- or two-sided<br>*Only common tests should be described solely by name; describe more complex techniques in the Methods section.*
☐	☒	A description of all covariates tested
☐	☒	A description of any assumptions or corrections, such as tests of normality and adjustment for multiple comparisons
☐	☒	A full description of the statistical parameters including central tendency (e.g. means) or other basic estimates (e.g. regression coefficient) AND variation (e.g. standard deviation) or associated estimates of uncertainty (e.g. confidence intervals)
☐	☒	For null hypothesis testing, the test statistic (e.g. F, t, r) with confidence intervals, effect sizes, degrees of freedom and P value noted<br>*Give P values as exact values whenever suitable.*
☒	☐	For Bayesian analysis, information on the choice of priors and Markov chain Monte Carlo settings
☒	☐	For hierarchical and complex designs, identification of the appropriate level for tests and full reporting of outcomes
☐	☒	Estimates of effect sizes (e.g. Cohen's d, Pearson's r), indicating how they were calculated

Our web collection on statistics for biologists contains articles on many of the points above.

Software and code

Policy information about availability of computer code

Data collection	The software and code used for data collection are listed below. Each was used as described in the manuscript.<br><br>-FACSDiva v8.0.1
Data analysis	The software and code used to analyze the data are listed below. Each was used as described in the manuscript.<br><br>-FloJo v10.8.1<br>-DESeq2 v1.22.2<br>-limma v3.38.3, v3.40.6<br>-enrichR v2.1<br>-Cutadapt v1.18<br>-STAR v2.7.1a<br>-Drop-seq v1.12<br>-Seurat2,<br>-Cell Ranger v3.0.2<br>-LOUPE v3.0.1<br>-MAST v1.8.2<br>-Monocle3<br>-ChIPseeker v1.32<br>-bowtie2<br>-Samtools v1.9<br>-Picard Tools v2.20.2

-Macs2
-Bedtools v2.27.1
-Homer v4.9.1
-SCENIC v 1.1.2-2
-GENIE3 v1.4.0
-PRISM v9
-R v3.5.1

For manuscripts utilizing custom algorithms or software that are central to the research but not yet described in published literature, software must be made available to editors and reviewers. We strongly encourage code deposition in a community repository (e.g. GitHub). See the Nature Research guidelines for submitting code & software for further information.

Data

Policy information about availability of data

All manuscripts must include a data availability statement. This statement should provide the following information, where applicable:
- Accession codes, unique identifiers, or web links for publicly available datasets
- A list of figures that have associated raw data
- A description of any restrictions on data availability

All data are available on GEO under the accession number GSE142215. This data has been made public and is available at: https://www.ncbi.nlm.nih.gov/geo/query/acc.cgi?acc=GSE142215 . Figures 1 to 5 and S1 and S10 have associated raw data available via GSE142215. There are no restrictions on availability of the data.

To analyze these data, we used Mus musculus genome assembly GRCm38 mm10 (https://www.ncbi.nlm.nih.gov/assembly/GCF_000001635.20/), the comprehensive GRCm38 GENCODE gene annotation (https://www.gencodegenes.org/mouse/release_M2.html) and Cyclebase (https://cyclebase.org/CyclebaseSearch).

Field-specific reporting

Please select the one below that is the best fit for your research. If you are not sure, read the appropriate sections before making your selection.

[x] Life sciences [ ] Behavioural & social sciences [ ] Ecological, evolutionary & environmental sciences

For a reference copy of the document with all sections, see nature.com/documents/nr-reporting-summary-flat.pdf

Life sciences study design

All studies must disclose on these points even when the disclosure is negative.

Sample size	Sample sizes are indicated in the figure legends. Sample sizes were based on previous experiments or pilot experiments. The sizes were chosen to enable comparisons of interest, for example between samples with different genotypes, at different stages or from different lineages. The metrics used to evaluate reproducibility and make statistical comparisons between the groups are detailed in the figure captions and methods of the manuscript.
Data exclusions	All data were included with the exception of outliers in the 3' end count libraries of phase-sorted embryos between E7.5 and E9.5 We used global UMAPs to quality control of the mRNA-seq samples, where the expectation is that samples of the same embryonic stage would cluster together. The samples that did not cluster by stage were removed. Inspection of the expression profiles suggests that the failure of these samples to cluster with samples of the same group was likely due to contaminating yolk sac or poor overall library quality.<br><br>We also excluded a comparison of phasic expression between wt and ccne1sd mutant cells during neural differentiation because the wt cells had differentiated to the point where the data was heavily confounded by expression of genes from postmitotic cells
Replication	All attempts at replication were successful.<br><br>Distinct embryos were used for replicates in all cases except the single-cell RNA-seq knockout experiments (Figure 4) where only a single capture was performed for each of mir-302-/- and p27-/-. Notably, the single-cell RNA-seq experiments on knockout embryos were a complimentary approach to the bulk RNA-seq on the same mutants. In the single-cell experiments, cellular permutations were used as internal replicates for the experiments (see methods).<br><br>Aside from the single-cell experiments on mutant embryos, each experiment was replicated at least twice. The exact number of replications for each experiment is provided in the associated caption.
Randomization	Groups were designated based on embryonic stages (Downs and Davies, 1993), cell cycle phases (gating of Hoechst staining), lineages (defined by expression) and genotypes. Additional detail on each grouping is provided in the manuscript.
Blinding	The majority of studies were not blinded as the samples were primarily handled by an individual experimentalist. Quantification of the overlap in Cyclin E1 and Cyclin B1 protein in E7.5, E8.5 and E9.5 was performed by an individual blinded to the remainder of this study.

Reporting for specific materials, systems and methods

We require information from authors about some types of materials, experimental systems and methods used in many studies. Here, indicate whether each material, system or method listed is relevant to your study. If you are not sure if a list item applies to your research, read the appropriate section before selecting a response.

Materials & experimental systems

n/a	Involved in the study
☐	☒ Antibodies
☐	☒ Eukaryotic cell lines
☒	☐ Palaeontology and archaeology
☐	☒ Animals and other organisms
☒	☐ Human research participants
☒	☐ Clinical data
☒	☐ Dual use research of concern

Methods

n/a	Involved in the study
☒	☐ ChIP-seq
☐	☒ Flow cytometry
☒	☐ MRI-based neuroimaging

Antibodies

Antibodies used

Primary: Chicken anti-GFP (1:200, Aves Labs, GFP-1010), OCT4 at 1:200 (BD Biosciences, 611202), NANOG at 1:200 (Abcam, ab21603), pRb 807/811 PE-conjugated antibody (Cell Signaling Technologies, D20B12), TUJ1 at 1:1000 (Abcam, ab18207), Cyclin E1 for IF at 1:200 (R&D Systems, AF6810), Cyclin B1 at 1:200 (Cell Systems Technology, 4138), Cyclin E1 for flow at 1:100 (Cell Signaling Technologies, 20808S)

Secondary: Alexa Fluor 488 Donkey Anti-Chicken IgY (Jackson ImmunoResearch, 703-546-155), Alexa Fluor 594 Goat^Mouse (ThermoFisher, A21125), Alexa Fluor 488 Donkey^Rabbit IgG (ThermoFisher, A21206), Alexa Fluor 594 Goat^Rabbit (Invitrogen, A11012) and Alexa Fluor 488 Goat^Rabbit (Invitrogen, A32731TR), Alexa Fluor 488 Goat^Mouse IgG1 (Jackson ImmunoResearch, 115-545-205), Alexa Fluor 594 Donkey^Sheep IgG (ThermoFisher, A11016).

Validation

GFP: GFP was evaluated in mir-302 GFP-knock-in reporter animals relative to wt embryos. The observed distribution is consistent with the distribution evident in fresh samples. Please see the manufacturer's website for additional validation and associated citations: https://www.aveslabs.com/products/anti-green-fluorescent-protein-antibody-gfp

OCT4/NANOG: Both were evaluated in mouse embryonic stem cells for positive staining, and compared to mouse embryonic fibroblast and neuroepithelium as negative controls. Please see the manufacturer's website for additional validation and associated citations: https://www.bdbiosciences.com/en-us/products/reagents/microscopy-imaging-reagents/immunofluorescence-reagents/purified-mouse-anti-oct3-4.611202 (OCT4), and https://www.abcam.com/nanog-antibody-ab80892.html (NANOG).

pRb: Evaluated by flow in mouse embryonic stem cells using an Rb knockout line as a control. Also as expected, pRb also increases following G1/S transit. Please see the manufacturer's website for additional validation and associated citations: https://www.cellsignal.com/products/antibody-conjugates/phospho-rb-ser807-811-d20b12-xp-rabbit-mab-pe-conjugate/11917

TUJ1: Used embryonic sections with postmitotic neurons as well as in vitro differentiated neurons as positive controls. ES cells and non-neuronal embryonic regions served as negative controls. Please see the manufacturer's website for additional validation and associated citations: https://www.abcam.com/beta-iii-tubulin-antibody-neuronal-marker-ab18207.html

Cyclin E1: Compared positive signal in mitotic cells to negative signal in post-mitotic cells. Please see the manufacturer's website for additional validation and associated citations: https://www.rndsystems.com/products/human-cyclin-e1-antibody_af6810 (IF), and https://www.cellsignal.com/products/primary-antibodies/cyclin-e1-d7t3u-rabbit-mab/20808 (Flow)

Cyclin B1: Evaluated cellular distribution (nuclear in M phase and cytoplasmic in other cell cycle phases). Please see the manufacturer's website for additional validation and associated citations: https://www.cellsignal.com/products/primary-antibodies/cyclin-b1-antibody/4138

Eukaryotic cell lines

Policy information about cell lines

Cell line source(s)

V6.5 embryonic stem (ES) cells (Jaenisch lab); DGCR8-/- ES cells (derived in house in the Blelloch lab); SBR reporter ES cells (Suter lab). NIH/3T3 fibroblasts (ATCC). Novel mutants generated as described in methods.

Authentication

The DGCR8-/- ES cells were generated in the Blelloch lab and genotyped by southern and western blots.

The SBR ESCs were generated in the Suter lab, and authenticated by genotyping (PCR) and using directed differentiations.

The 3T3 cells were authenticated via morphology.

	The novel mutants were authenticated as described in the manuscript.
Mycoplasma contamination	All ES and 3T3 lines were tested for mycoplasma before use. Active cell lines in the Blelloch lab are also spot-checked annually or more frequently.
Commonly misidentified lines (See ICLAC register)	No commonly misidentified cell lines were used in this study.

Animals and other organisms

Policy information about studies involving animals; ARRIVE guidelines recommended for reporting animal research

Laboratory animals	species: mus musculus, strain: C57Bl6/J, males and females. Adult animals >6 weeks of age were bred and embryos analyzed at the stages described throughout the manuscript.<br><br>Mice were maintained on a 12 hour light/dark cycle. Mice were bred as trios and housed 5 per cage otherwise. Mice were housed between 20 and 23 degrees Celsius and 40-50% relative humidity.
Wild animals	no wild animals were used in this study
Field-collected samples	no field collected samples were used in this study
Ethics oversight	All mice were maintained in accordance with the UCSF animal husbandry guidelines. All experiments were reviewed and approved by the UCSF Animal Care and Use Committee.

Note that full information on the approval of the study protocol must also be provided in the manuscript.

Flow Cytometry

Plots

Confirm that:

- [x] The axis labels state the marker and fluorochrome used (e.g. CD4-FITC).
- [x] The axis scales are clearly visible. Include numbers along axes only for bottom left plot of group (a 'group' is an analysis of identical markers).
- [x] All plots are contour plots with outliers or pseudocolor plots.
- [x] A numerical value for number of cells or percentage (with statistics) is provided.

Methodology

Sample preparation	Detailed in the methods section of the manuscript.
Instrument	Flow cytometry was performed on BD LSR II instruments. FACS was performed on BD FACSAria II instruments.
Software	FACSDiva software was used to collect the data and FloJo v10.6.1 was used to analyze the data.
Cell population abundance	The abundance of relevant populations is quantified and presented throughout the manuscript. Re-sorting of gated collected fractions was used during the initial FACS quality control to evaluate protocols. Additional purity metrics of the transcription-profiled single-cells are provided throughout the manuscript.
Gating strategy	The following gating strategy was used: 1) Cells from culture or dissociated embryos were sorted from debris based on FSC/SSC gates, 2) Single cells were separated from doublets based on concordance of FSC-A and FSC-H, and 3) Phases of the cell cycle were sorted based on gating of gap phases. Positive cells were defined by negative cellular controls: e.g. mir-302-GFP+ by mir-302-GFP- embryos, Sox1+/Bry+ by wild type ESCs and pRb+ relative to Rb-/- ESCs.

- [x] Tick this box to confirm that a figure exemplifying the gating strategy is provided in the Supplementary Information.
